# Coral microbiome diversity reflects mass coral bleaching susceptibility during the 2016 El Niño heat wave

**DOI:** 10.1002/ece3.4662

**Published:** 2019-01-17

**Authors:** Stephanie G. Gardner, Emma F. Camp, David J. Smith, Tim Kahlke, Eslam O. Osman, Gilberte Gendron, Benjamin C. C. Hume, Claudia Pogoreutz, Christian R. Voolstra, David J. Suggett

**Affiliations:** ^1^ University of Technology Sydney Climate Change Cluster Ultimo NSW 2007 Australia; ^2^ Coral Reef Research Unit, School of Biological Sciences University of Essex Colchester UK; ^3^ Marine Biology Department, Faculty of Science Al‐Azhar University Cairo Egypt; ^4^ Seychelles National Parks Authority Victoria Seychelles; ^5^ Red Sea Research Center, Biological and Environmental Sciences and Engineering Division (BESE) King Abdullah University of Science and Technology (KAUST) Thuwal Saudi Arabia

**Keywords:** bacterial community composition, coral bleaching, microbiome, Seychelles, *Symbiodiniaceae*

## Abstract

Repeat marine heat wave‐induced mass coral bleaching has decimated reefs in Seychelles for 35 years, but how coral‐associated microbial diversity (microalgal endosymbionts of the family Symbiodiniaceae and bacterial communities) potentially underpins broad‐scale bleaching dynamics remains unknown. We assessed microbiome composition during the 2016 heat wave peak at two contrasting reef sites (clear vs. turbid) in Seychelles, for key coral species considered bleaching sensitive (*Acropora muricata*,* Acropora gemmifera*) or tolerant (*Porites lutea*,* Coelastrea aspera*). For all species and sites, we sampled bleached versus unbleached colonies to examine how microbiomes align with heat stress susceptibility. Over 30% of all corals bleached in 2016, half of which were from *Acropora* sp. and *Pocillopora* sp. mass bleaching that largely transitioned to mortality by 2017. Symbiodiniaceae ITS2‐sequencing revealed that the two *Acropora* sp. and *P. lutea* generally associated with C3z/C3 and C15 types, respectively, whereas *C. aspera* exhibited a plastic association with multiple D types and two C3z types. 16S rRNA gene sequencing revealed that bacterial communities were coral host‐specific, largely through differences in the most abundant families, Hahellaceae (comprising *Endozoicomonas*), Rhodospirillaceae, and Rhodobacteraceae. Both *Acropora* sp. exhibited lower bacterial diversity, species richness, and community evenness compared to more bleaching‐resistant *P. lutea* and *C. aspera*. Different bleaching susceptibility among coral species was thus consistent with distinct microbiome community profiles. These profiles were conserved across bleached and unbleached colonies of all coral species. As this pattern could also reflect a parallel response of the microbiome to environmental changes, the detailed functional associations will need to be determined in future studies. Further understanding such microbiome‐environmental interactions is likely critical to target more effective management within oceanically isolated reefs of Seychelles.

## 1 INTRODUCTION

Coral reef ecosystems are exceptionally vulnerable to anthropogenic disturbance and have been decimated by climate change‐driven marine heat waves during 2015–2017, with >30% of all corals lost at many locations worldwide through bleaching (Hughes et al., 2018, 2017). Western Indian Ocean (WIO) reefs were particularly affected by Sea Surface Temperature (SST) anomalies throughout 2016 broadly exceeding 10–15 maximum degree heating weeks (DHW), driving severe bleaching and mortality throughout this region (Hughes et al., 2018). Reefs within the WIO have in fact been repeatedly impacted by heat waves throughout the last 20 years (Graham, Jennings, MacNeil, Mouillot, & Wilson, 2015; Hughes et al., 2018; McClanahan, Ateweberhan, Darling, Graham, & Muthiga, 2014), and the combination of smaller scale thermal anomalies with other stressors has increasingly limited long‐term coral recovery (e.g., Graham et al., 2015; Zinke et al., 2018).

Seychelles coral reefs were among the most impacted globally during the 1998 mass bleaching, with coral cover reduced by >90% across the inner islands (Graham et al., 2006; Wilson et al., 2012). Recovery has been limited by strong phase shifts toward algal dominance in several reefs (Graham et al., 2015; Wilson et al., 2012), and recruitment bottlenecks (Chong‐Seng, Graham, & Pratchett, 2014), such that coral cover and diversity in Seychelles is now lower than for many other regions in the WIO (Harris, Wilson, Graham, & Sheppard, 2014). Reefs are characterized by patch, granitic, and carbonaceous habitats across coastal fringing and oceanic environments (Graham et al., 2006; Jennings, Grandcourt, & Polunin, 1995). However, loss of coral cover and diversity since 1998 has been greatest for the carbonaceous reefs (Graham et al., 2008; Wilson et al., 2012), resulting in reduced carbonate budgets, accretion potential, and structural maintenance (Januchowski‐Hartley, Graham, Wilson, Jennings, & Perry, 2017) that underpin the critical ecosystem service value of Seychelles’ reefs (Clifton et al., 2012). Existence of functional diversity at macroecological scales of lower latitude reefs in the WIO, including Seychelles, has recently been identified as a key determinant of longer‐term resilience to environmental stress (Zinke et al., 2018). However, such a role for microecological processes in the WIO remains largely unknown, in particular microbial community composition and functioning that can be critical in determining coral stress resilience in other reef regions worldwide (Putnam, Barott, Ainsworth, & Gates, 2017; Suggett, Warner, & Leggat, 2017).

Various components of the coral holobiont, that is, the cnidarian host and its microbial community (the “microbiome”), play key roles in regulating corals’ resistance to heat stress (Morrow, Muller, & Lesser, 2018; Sweet & Bulling, 2017). Changes in phylogenetic diversity of the endosymbiont (*family* Symbiodiniaceae, formerly genus *Symbiodinium*; LaJeunesse et al., 2018) are well described to correspond with bleaching susceptibility (Suggett et al., 2017), whereby corals often associate with known heat‐tolerant Symbiodiniaceae taxa (e.g., Howells et al., 2012; Hume et al., 2015; Pettay, Wham, Smith, Iglesias‐Prieto, & LaJeunesse, 2015; Ziegler, Eguíluz, Duarte, & Voolstra, 2017a). However, the coral holobiont also hosts a highly diverse bacterial community (Bourne & Munn, 2005; Frias‐Lopez, Zerkle, Bonheyo, & Fouke, 2002; Rohwer, Breitbart, Jara, Azam, & Knowlton, 2001; Rohwer, Seguritan, Azam, & Knowlton, 2002), which in some cases form species‐specific associations with corals (Neave, Rachmawati, et al., 2017a; Rohwer et al., 2001, 2002 ). Microbial communities may facilitate acclimatization of the coral holobiont to changes in the environment through rapid restructuring of the community (Reshef, Koren, Loya, Zilber‐Rosenberg, & Rosenberg, 2006; Torda et al., 2017; Ziegler, Seneca, Yum, Palumbi, & Voolstra, 2017b), and studies indicate that an intact (Krediet, Ritchie, Paul, & Teplitski, 2013; Roder, Bayer, Aranda, Kruse, & Voolstra, 2015; Rosenberg, Koren, Reshef, Efrony, & Zilber‐Rosenberg, 2007) and diverse (Hadaidi et al., 2017) coral microbiome may be essential to coral immunity and health. Responses of coral‐associated bacterial communities to shifts in coral health (Bourne, Iida, Uthicke, & Smith‐Keune, 2008; Cárdenas, Rodriguez‐R, Pizarro, Cadavid, & Arévalo‐Ferro, 2012; Glasl, Herndl, & Frade, 2016; Guest et al., 2016; Jones, Berkelmans, Oppen, Mieog, & Sinclair, 2008; Ziegler, Seneca et al., 2017b) and environmental stressors (e.g., Garren, Son, Tout, Seymour, & Stocker, 2015; Jessen et al., 2013; Kwiatkowski, Cox, Halloran, Mumby, & Wiltshire, 2015; Vega Thurber et al., 2009; Vega Thurber et al., 2014) have been extensively explored and reported, with recent evidence suggesting flexibility of these communities may determine holobiont resistance to environmental stress (Pogoreutz et al., 2018).

**Figure 1:**
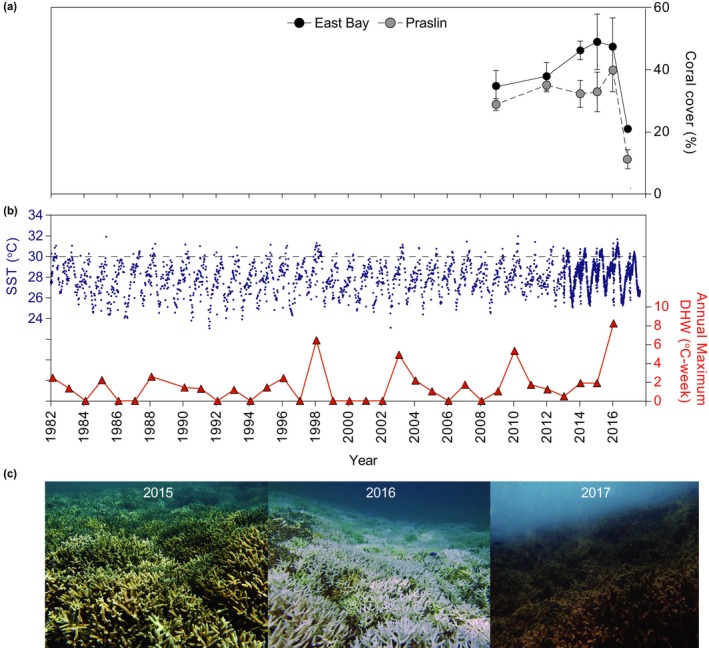
(a) Coral cover for East Bay (black symbols) and Praslin (light‐gray symbols), (b) sea surface temperatures (SST) between 1982 and 2017 for the Seychelles (dark blue symbols) showing the annual maximum degree heating weeks (DHW; red triangles). Dotted line indicates the average sea surface temperature recorded for Seychelles. Averages ±SE shown for coral cover (*n* = 3 for East Bay and 6 for Praslin). (c) Photographs showing coral cover and condition between 2015 and 2017 for *Acropora* sp.

Microbiome composition is important in determining coral health over space and time, yet is completely undescribed for the coral communities in Seychelles. Therefore, as part of a long‐term program examining carbonaceous coral communities within Curieuse Marine National Park (Seychelles), we evaluated Symbiodiniaceae and bacterial diversity and community composition among key reef‐building coral species (*Acropora gemmifera*,* Acropora muricata*,* Coelastrea aspera*, and *Porites lutea*) and for populations within a clear water versus turbid reef environment. Importantly, this combination of species included those previously described as either heat stress sensitive (*Acropora* spp.) or tolerant (*C*. *aspera*,* P. lutea*) in the Seychelles (Harris et al., 2014; Smith, Wirshing, Baker, & Birkeland, 2008) and wider WIO (McClanahan et al., 2014, 2007). Microbial sampling coincided with the 2016 mass bleaching marine heat wave in April 2016 (Figure 1) enabling us to uniquely differentiate microbiomes for bleached versus unbleached colonies within these two environments. In doing so, we show for the first time how microbial signatures vary during chronic heat exposure across complex species‐environment interactions in the WIO. Microbiomes were generally conserved among bleached versus unbleached colonies for the two species of *Acropora* sp. suggesting small‐scale environmental variability (e.g., shading from surrounding substrates, sensu Hoogenboom et al., 2017) are likely critical in ensuring persistence of heat stress‐sensitive corals in oceanically isolated reef systems such as Seychelles.

## 2 MATERIALS AND METHODS

### 2.1 Site description and benthic sampling

Coral communities have been examined from two fringing carbonaceous reef sites within Curieuse Marine National Park (CMNP) since 2009 (Supporting Information Figure S1): A relatively clear water reef along the southern coast of Curieuse Island (East Bay; 4°16′55″S 55°44′32″E) and a relatively turbid reef on the northern coast of Praslin Island (Praslin; 4°18′35″S 55°43′28″E). Both sites are subject to a semidiurnal tidal cycle, with mean ± *SE* tidal range of 1.3 ± 0.2 m and assessed annually (2009–2017) via 2‐week sampling campaigns to coincide with the end of the northwest monsoon wind season when SST is warmest (April–May). In 2016, this campaign coincided with the peak of the prolonged global heatwave, where cumulative heat stress for Seychelles exceeded and 10 DHW (^o^C‐weeks; https://coralreefwatch.noaa.gov/satellite/index.php; Mahe). Reefs within this region were on “Bleaching Watch” from November 2015 and “Alert Level 2” by April 2016.

Coral cover and species identification were determined for all years, and sampling for Symbiodiniaceae and bacterial community diversity was conducted in 2016. Coral cover data are presented as means ± standard error (*SE*).

### 2.2 Abiotic measurements

To assess the historical thermal stress specifically around CMNP, remotely sensed Sea Surface Temperatures (SST) and Degree Heating Weeks (DHWs) were extracted for Curieuse Island (lat −4.297764, lon 55.727171) for the last 35 years (1982–2017) from two high‐resolution sources: (a) weekly CoRTAD data (Pathfinder 5.2, AVHAR, 4 km resolution) (https://data.nodc.noaa.gov/cgi-bin/iso?xml:id=gov.noaa.nodc:0126774) for the period between 1982 and 2012 (Casey et al., 2015); and (b) daily Coral Reef Watch (CRW; 5 km resolution) data for the period from 2013 to 2017 (Liu et al., 2014). Data were extracted from single grid (pixel) avoiding land interference using ArcGIS 10.3.1 software. In addition, temperature was measured at each location over the two‐week sampling period using calibrated HOBO® pendant loggers set at 20‐s measuring intervals, to verify local conditions relative to the global SST retrieval, as well as contrast SSTs on the reef flat (1–2 m) versus upper slope (5–8 m). Consistent with the remotely sensed SSTs (Figure 1b), maximum daily SSTs throughout our 2016 sampling period exceeded 32ºC, ranging 32.3–32.9ºC and 31.8–32.3ºC at 1–2 m and 5–8 m, respectively. Light was measured at two depths (6 and 1 m) using intercalibrated (no breaking) HOBO® pendant light loggers in 2014–2015. Data were converted from Lux to µmol photons m^−2^ s^−1^ (as per Long, Rheuban, Berg, & Zieman, 2012) to return light attenuation coefficients (*K*_D_ (PAR), m^−1^) for each site. As expected, *K*_D_ (PAR) was highly variable at both sites as a result of tidal resuspension of sediment, but the hourly integrated range for clear water East Bay (0.14–0.21 m^−1^; *n* = 83) generally was half that for turbid Praslin (0.23–0.38 m^−1^; *n* = 59). Previous work at these sites has demonstrated that pH, *A*_T_, salinity, PCO_2_, and nitrate concentrations are similar between locations for this time of year, ca. 8.12 (total scale), 2.360 μmol kg/SW, 35.5 ppm, 323 μatm, and 1.1 μM, respectively (for details, see Camp et al., 2016).

### 2.3 Characterizing benthic habitat

Standard continuous line intercept transects were conducted using SCUBA diving and were used to quantify coral community structure at these sites. Data were recorded by high‐definition video (*Sony* HDR‐SR1E, 2009; *Canon* G10, 2011–2013; *GoPro Hero series* from 2014–2017) and later analyzed to determine habitat type as percentage live coral cover to species level. Both sites were characterized by a shallow (1–3 m) reef flat that transitioned to a slope of semicontinuous consolidated carbonaceous reef to depths of 12–13 m (Praslin) or 10–14 m (East Bay). Importantly, these reef flats were very different habitat types at the two sites, specifically patch reef and sand at Praslin versus rubble at East Bay; thus, we restricted our analysis to the upper reef slope to standardize habitat type, performed as 3 × 30 m (2009, 2012, 2014–2017) continuous line intercept transects (5–8 m depth) at each site per year. All coral data from 2016 were further categorized to account for bleaching intensity. Our initial categorization adopted that of McClanahan et al. (2007), which assigns a score based on % live coral surface area fully bleached. However, since all observations during the mass bleaching were 80%–100% of the live coral surface area fully bleached, we eventually defined categories as unbleached, bleached (pale and with host tissue remaining), and recently dead (host tissue lost and/or first signs of algal overgrowth).

### 2.4 Microbiome sampling and DNA extraction

All colonies were sampled 6–7 days after strong bleaching was first observed. Triplicate fragments were taken from independent colonies for each of the four key coral species found throughout the upper reef slope at both turbid (Praslin) and clear water (East Bay) sites: *A. gemmifera, A. muricata, P. lutea,* and *C. aspera* (three fragments × four species × two sites × two coral condition = 48 fragments total). Replicate colonies of the same species were generally sampled >20 m apart, although unbleached colonies were often within close proximity (5–10 m) of neighboring bleached colonies. A small portion of each fragment was removed for Symbiodiniaceae cell density assessment using a haemocytometer and normalized to surface area (as per Camp et al., 2016). The remaining fragment was immediately preserved in RNAlater for bacterial 16S and Symbiodiniaceae ITS2 rRNA genotyping and stored at −80°C until processing.

For DNA extraction, coral fragments were thawed slowly on ice and removed from the RNAlater solution using sterile forceps and kimwipes to remove excess RNAlater (Tout et al., 2015; Vega Thurber et al., 2009). Coral fragments were transferred into sterile ziplock bags and mucus and tissues subsequently air‐blasted using airflow from a sterile pipette tip (1,000 µl filter barrier tips; Neptune, USA) into 5 ml PBS‐EDTA. Care was taken to exclude any skeletal fragments (to reduce contamination by the endolithic microbiome). DNA was extracted using the Qiagen DNeasy Plant Mini Kit (Qiagen, Hilden, Germany) according to manufacturer's instructions. To disrupt Symbiodiniaceae cells, 200 µl tissue slurry in 200 µl AP‐1, 4 µl RNase A stock solution (100 g/ml), and 200 µl 0.5 mm sterile glass beads (BioSpec, Bartlesville, OK, USA) were bead‐beaten at 30 Hz for 90 s with a Tissue Lyser II (Qiagen). An additional step using phenol:chloroform:isoamyl alcohol (25:24:1) prebuffered to pH 8 was used to purify the DNA. Mock samples (i.e., empty) were used for DNA extraction to account for kit contaminants (Pogoreutz et al., 2018; Salter et al. 2014). Extracted DNA was quantified and quality checked using a NanoDrop 2000C spectrophotometer (Thermo Fisher Scientific, Waltham, MA, USA). PCR amplifications (including mock [i.e., no template] PCRs) to account for contaminants (Pogoreutz et al., 2018; Salter *et al*. 2014) were performed in triplicate reactions with Qiagen Multiplex PCR Kit (Qiagen; see below).

### 2.5 PCR amplification and sequencing

For Symbiodiniaceae typing, amplification of the ribosomal Internal Transcribed Spacer 2 (ITS2) region, a multicopy genetic marker commonly used to assess Symbiodiniaceae diversity was used (Arif et al., 2014; Smith, Ketchum, et al., 2017; Smith, Vaughan, Ketchum, McParland, & Burt, 2017). For amplicon‐specific PCRs, we used the primers ITSintfor2 5'‐TCG TCG GCA GCG TCA GAT GTG TAT AAG AGA CAG GAA TTG CAG AAC TCC GTG‐3' and ITS2‐reverse 5'‐GTC TCG TGG GCT CGG AGA TGT GTA TAA GAG ACA GGG GAT CCA TAT GCT TAA GTT CAG CGG GT‐3' with concatenated Illumina sequencing adapters (underlined). Initial PCR amplification was achieved with the following thermal cycles: 94°C for 15 min, then 35 cycles of 94°C for 30 s, 51°C for 30 s, 72°C for 30 s, followed by one cycle of 72°C for 10 min and 4°C hold (Coleman, Suarez, & Goff, 1994; LaJeunesse, 2002).

To amplify the bacterial 16S rRNA gene, we used the primers 16SMiSeqF‐Andersson 5‐TCGTCGGCAGCGTCAGATGTGTATAAGAGACAGAGGATTAGATACCCTGGTA‐3′ and 16SMiSeqR‐Andersson 5′‐GTCTCGTGGGCTCGGAGATGTGTATAAGAGACAGCRRCACGAGCTGACGAC‐3´ (Illumina sequencing adapters underlined) that target the variable regions five and six of the 16S gene (Andersson et al., 2008). These primers have previously been reported to amplify well with coral DNA (Bayer et al., 2013; Röthig, Ochsenkühn, Roik, Merwe, & Voolstra, 2016). The thermal conditions for 16S amplicon PCRs were as follows: 95°C for 15 min, followed by 27 cycles of 95°C for 40 s, 55°C for 40 s, 72°C for 40 s, and a final extension cycle of 72°C at 10 min (Ziegler et al., 2016).

For individual PCR reactions, DNA was aliquoted to 12–50 ng/L, with 10 µl Qiagen Mix, 0.5 µl of each 10 M primer mix, 1 µl of DNA template, and RNAse‐free water to adjust the reaction volume to 20 µl. 10 µl of each PCR product was run on an 1% agarose gel to visualize successful amplification.

Sample triplicates were subsequently pooled and then purified using the Agencourt AMPure XP magnetic bead system (Beckman Coulter, Brea, CA, USA). Purified PCR products were subjected to an indexing PCR (eight cycles) to add Nextera XT indexing and sequencing adapters (Illumina) according to the manufacturer's protocol. Indexed amplicons were again purified, quantified on the QuBit (Quant‐IT dsDNA Broad Range Assay Kit; Invitrogen, Carlsbad, CA, USA) and pooled in equimolar ratios on the BioAnalyzer (Agilent Technologies, Santa Clara, CA, USA). The final pooled library was purified on a 2% agarose gel to remove excess primer dimer. The library was sequenced at 8 p.m. with 10% phiX on the Illumina Miseq, 2× 300 bp end version 3 chemistry according to the manufacturer's specifications at the Bioscience Core Lab (KAUST, Saudi Arabia).

### 2.6 Microbial analysis—Symbiodiniaceae

The SymPortal analytical framework (symportal.org, github.com/SymPortal) was used to predict putative Symbiodiniaceae taxa. Briefly, the multicopy nature of the rRNA gene means that every Symbiodiniaceae genome contains hundreds to thousands of copies of it. Each of these gene copies is able to accrue mutations somewhat independently. As such, considerable intragenomic sequence diversity that may be leveraged for purposes of taxonomic delineation is found within every Symbiodiniaceae cell (Hume, D'Angelo, Burt, & Wiedenmann, 2018). SymPortal aims to make use of this diversity, using next‐generation amplicon sequencing data, to resolve between genetically differentiated taxa. SymPortal works by identifying specific sets of defining intragenomic ITS2 sequence variants (DIVs) that are used to define the taxonomic unit of SymPortal, the ITS2 type profile, indicative of genetically differentiated Symbiodiniaceae taxa. Demultiplexed and paired forward and reverse fastq.gz files outputted from the Illumina sequencing were submitted directly to SymPortal. Sequence quality control was conducted as part of the SymPortal pipeline using Mothur 1.39.5 (Schloss et al., 2009), the BLAST + suite of executables (Camacho et al., 2009), and minimum entropy decomposition (MED; Eren et al., 2015).

Over the years, development and variation in the range of techniques employed to genetically differentiate within the Symbiodiniaceae have led to a range of different terms being used to describe the genotypic units of resolution. For example, “type,” “ITS2 type,” “ITS2 profile,” “ITS2 fingerprint,” “clade,” “subclade,” “subtype,” and most recently “ITS2 type profile” are among the most common. Some of these are used interchangeably in one setting, while representing different entities in others. To clarify and limit ambiguity, for the purposes of this study, we will restrict our use to “type” and “ITS2 type profile.” A type refers to Symbiodiniacea taxa that have a specific sequence or set of sequences as their most abundant sequence. An ITS2 type profile is a set of sequences that are used to define either a putative or defined taxa. For example, *Durusdinium trenchii* is a D1 type and has an ITS2 type profile of D1–D4.

### 2.7 Microbial analysis—bacteria

Sequencing reads for 16S bacterial analysis were processed as outlined in Kahlke (2018). In summary, sequences were joined using FLASH (Magoč & Salzberg, 2011) and subsequently trimmed using MOTHUR (Parameters: minlength = 294, maxlength = 301, maxhomop = 6, maxambig = 0). The resulting fragments were clustered into operational taxonomic units (OTUs) and identity threshold of 97% and chimeric sequences were identified using VSEARCH (Rognes, Flouri, Nichols, Quince, & Mahé, 2016) and the SILVA v128 database. Unwanted sequences related to Archaea, mitochondria, chloroplasts, and kit contaminants (confirmed with sequencing of mock samples and PCRs; OTUs 2, 4, and 7; *Brevibacter casei*,* Shigella flexneri*, and *Brachybacterium* sp., respectively) were removed. To assign taxonomy, QIIME (Caporaso et al., 2010) was used with the BLAST algorithm against the SILVA v128 database at a 97% similarity cutoff. Data were rarefied to 1,000 sequences per sample and the subsequent.biom file was used for all downstream applications. Alpha diversity indices (Chao1 index, Simpson's evenness, Shannon diversity index, and Phylogenetic diversity) were computed using QIIME.

### 2.8 Data analysis

To initially examine trends of total live coral cover, we pooled data according to species of *Acropora* and *Pocillopora* versus “other species” since, as in previous bleaching episodes in Seychelles (e.g., see Wilson et al., 2012), these two genera are typically most susceptible to bleaching. Unpaired *t*‐tests were used to analyze the percent of coral cover data, using Welch's correction for variable standard deviations (GraphPad Prism v.6).

A univariate general linear model (GLM) for bacterial alpha diversity indices (including number of reads, OTUs per sample, phylogenetic diversity, Chao1, Simpson's evenness, and Shannon diversity index) was used to compare interactive effects of species, site, and coral condition and a one‐way ANOVA used to detect significant differences within species using IBM SPSS Statistics (v.21; IBM Corporation, New York, NY, USA). Coral‐associated bacterial assemblages were tested for differences between species (*A. gemmifera, A. muricata, C. aspera,* and *P. lutea*), sites (East Bay and Praslin), and coral condition (bleached and unbleached) using permutation multivariate analysis of variance (PERMANOVA) at family level. Here, all fixed factors (sites and coral condition) were nested according to hierarchy, and 9,999 permutations of residuals were conducted based on Bray–Curtis distances between fourth‐root transformed samples using the PRIMER‐E software with the PERMANOVA+ add‐on package v1.0.6 (Anderson, Gorley, & Clarke, 2008; Clarke & Gorley, 2006). Analysis of Similarity (ANOSIM) was conducted for species, site, and coral condition, and a significant difference was only detected for species. Similarity percentage analysis (SIMPER; using species as the factor) revealed the main contributing bacterial families responsible for differences using PRIMER v6.1.16 (Clarke & Gorley, 2006). Analyses were conducted at family level (i.e., at a taxonomic level where the majority of OTUs are assigned) to observe differences that were not be resolved at the strain level. Beta diversity differences for bacterial community composition were visualized in a principal coordinate analysis (PCoA) based on a Bray‐Curtis dissimilarity matrix and Pearson's correlation. In addition, analysis of bacterial community data excluding the most abundant bacterial families (Hahellaceae, Rhodospirillaceae, and Rhodobacteraceae) were run using PERMANOVA to determine whether any community composition changes were masked by the dominant taxa (see Supporting Information).

Symbiodiniaceae diversity analysis was conducted using the ITS2 type profile data output by SymPortal. We tested for significant individual and interactive effects of species, site, and coral condition using permutational multivariate analysis of variance (PERMANOVA using the PRIMER‐E software with the PERMANOVA + add‐on package v1.0.6 (Anderson et al., 2008; Clarke & Gorley, 2006). ANOSIM was performed for species, site, and coral condition and a significant difference was only detected between species. SIMPER revealed the main contributing Symbiodiniaceae ITS2 type profile. Beta diversity was visualized in a principal coordinate analysis (PCoA) based on a Bray‐Curtis dissimilarity matrix and Pearson's correlation.

## 3 RESULTS

### 3.1 Impact of the 2016 heat wave event on live coral cover

Prior to 2016, Seychelles reefs experienced few years with prominent annual maximum degree heating weeks DHWs (>4°C‐weeks), notably, 1998, 2003, 2010 (see Figure 1b). In 2016, DHWs were the highest recorded for the last 35 years, where the annual maximum DHW in CMNP reached 8.2°C‐weeks (Figure 1b). By 2017, much of the live coral cover that had bleached in 2016 had transitioned to dead eroding coral covered by algal mats (Figure 1c).

**Figure 2:**
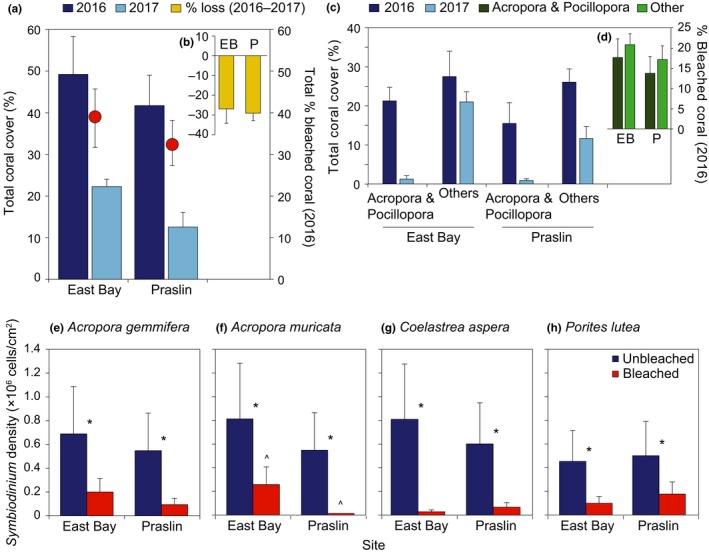
(a) Total coral cover for 2016 (dark blue bars) and 2017 (light blue bars; left *y*‐axis) and total percent of bleached coral in 2016 (red symbols; right *y*‐axis) and (b) total percent of coral lost between 2016 and 2017 (yellow bars) for East Bay (EB) and Praslin (P), (c) total coral cover for *Acropora* and *Pocillopora* and Other groups in 2016 (dark blue bars) and 2017 (light blue bars; left *y*‐axis) and (d) total percent of bleached coral in 2016 classified into *Acropora* and *Pocillopora* (dark green bars) and Other (light green bars; right *y*‐axis) groups for East Bay (EB) and Praslin (P). Averages ± *SE* are shown (*n* = 3 for East Bay and *n* = 6 for Praslin). Symbiodiniaceae cell density for unbleached (dark gray bars) and bleached (light gray bars) colonies of (e) *Acropora gemmifera*, (f) *Acropora muricata*, (g) *Coelastrea aspera*, and (h) *Porites lutea* at East Bay and Praslin. Asterisks (*) indicate significant differences between health, arrows (^) indicate a significant difference between site for the bleached colonies of *A. muricata* where *p* < 0.05. Data were log_10_ transformed for *A. muricata* and *P. lutea* and square‐root transformed for *C. aspera* for homogeneity of variance. Averages ± *SE* shown (*n* = 3, *n* = 2 for bleached *A. muricata* at Praslin)

Total live coral cover has been steadily increasing within CMNP at both sites between 2009 and 2016 (from 35% ± 5% to 49% ± 9% and from 30% ± 2% to 42% ± 7%, in East Bay and Praslin, respectively; Figure 1a). However, following the 2016 heat wave, total live coral cover at East Bay significantly declined from 49% ± 9% to 22% ± 2% (*t* = 2.91, *df *= 4, *p* < 0.05) and at Praslin from 42% ± 7% to 13% ± 3% (*t* = 3.61, *df *= 10, *p* < 0.005) between 2016 and 2017 (Figure 1a and 2a). Approximately one‐third of the coral cover identified at each site was bleached (39% ± 7% at East Bay and 32% ± 6% at Praslin, Figure 2a) at the peak of the heat wave during our sampling campaign in April 2016, resulting in a loss in coral cover of 27% ± 7% (East Bay) and 29% ± 4% (Praslin; Figure 2b).

Of the 49% total live cover at East Bay before the heat wave in 2016, almost half (21% ± 4%) was *Acropora* sp. and *Pocillopora* sp. with the remaining 28% ± 7% as other (Figure 2c; particularly *Porites* sp. but also *Montipora* sp. and *Favia* sp.). The large loss of coral cover by 2017 was attributed largely to mortality of *Acropora* sp. and *Pocillopora* sp., where live cover had declined to only 1% ± 1%, whereas all other taxa had declined to only 21% ± 3% (Figure 2c), despite no difference in the percent of bleached corals in 2016 for both sites (Figure 2d). Similar trends were observed at the more turbid Praslin site, where total live cover for *Acropora* sp. and *Pocillopora* sp. versus all other taxa was 16% ± 5% versus 26% ± 3% in 2016, declining to 0.9% ± 0.5% versus 12% ± 3% in 2017 (Figure 2c). As such, loss of taxa other than species of *Acropora* and *Pocillopora* was also important at this turbid site (and notably *Montipora* sp., *Lobophyllia* sp.).

### 3.2 Symbiodiniaceae communities

Symbiodiniaceae cell densities were variable across samples but overall lower for all bleached colonies (2 × 10^5^−1 × 10^4^ cm^−2^) compared to unbleached (8 × 10^7^−5 × 10^5^ cm^−2^) at both locations (see Figure 2e–h). Thus, substantial numbers of Symbiodiniaceae were still present in highly visibly bleached corals at the time of sampling in April 2016.

**Figure 3:**
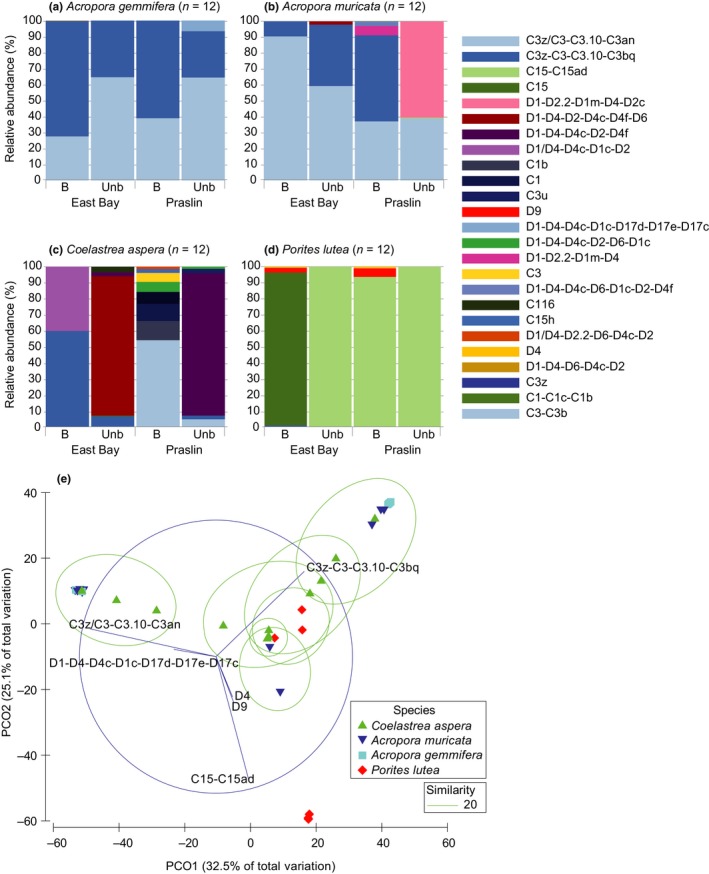
Average relative abundance (%) showing the Symbiodiniaceae ITS2 type profile for bleached and unbleached corals at East Bay and Praslin for (a) *Acropora gemmifera*, (b) *Acropora muricata*, (c) *Coelastrea aspera* and (d) *Porites lutea*. Colors represent different Symbiodiniaceae ITS2 type profiles. (e) Principle Coordinate Analysis (PCoA) for dominant Symbiodiniaceae ITS2 type profiles found in four coral species at East Bay and Praslin in Seychelles. Data was fourth‐root transformed, and a Bray‐Curtis similarity matrix was used with a correlation of 0.2. Ellipses denote similarity clusters of 20% (green dashed line). Percentages on axes indicate variation explained by the two coordinates

Symbiodiniaceae genera detected in 48 ITS2 gene libraries in all samples from the East Bay and Praslin sites were *Cladocopium* (formerly clade C; LaJeunesse et al., 2018) (13 different ITS2 type profiles, indicative of genetically differentiated *Cladocopium* spp. taxa) and *Durusdinium* (formerly clade D; 12 ITS2 type profiles, Figure 3a–d, Supporting Information Table S6). Among all hosts, the greatest Symbiodiniaceae diversity was observed for *C. aspera* (16 ITS2 type profiles identified) and the least for *A. gemmifera* (three identified). PERMANOVA revealed no significant interaction between species, site, and coral condition for all samples (pseudo *F*_3,47_ = 1.46, *p* = 0.164), although a significant difference between species was detected (pseudo *F*_3,47_ = 6.33, *p* < 0.001; Supporting Information Table S1a). Further analysis confirmed that no significant difference in Symbiodiniaceae ITS2 type profiles for site (ANOSIM; Global R = 0.01, *p* = 0.261) or coral condition (ANOSIM; Global R = 0.041, *p* = 0.073), and only significant differences between coral species detected (ANOSIM; Global R = 0.25, *p* < 0.0001; Supporting Information Table S1b), corresponding to distinct coral taxa specific responses: (a) *A. gemmifera*, generally showed little variance across sites or between coral condition (contribution from ITS2 type profiles of 51.96% from C3z‐C3‐C3.10‐C3bq and of 47.22% from C3z/C3‐C3.10‐C3an; Figure 3a, Supporting Information Table S2); (b) *A. muricata*, had different communities between unbleached sites (C3z/C3‐C3.10‐C3an for East Bay vs. D1‐D2.2‐D1m‐D4‐D2c dominant for Praslin) that then converged to a single “bleached” community of C3z/C3‐C3.10‐C3an and C3z‐C3‐C3.10‐C3bq for both sites (Figure 3b); (c) *C. aspera*, which had very different communities across site and bleached versus unbleached colonies, with the highest contribution from D1‐D4‐D4c‐D2‐D4f (28.62%) and 28.24% contribution from C3z/C3‐C3.10‐C3an (Figure 3c, Supporting Information Table S2) and finally (d) *P. lutea*, a single community of C15‐C15ad (91.61%) and C15 (5.75%) for both sites unbleached, but additions of D4 and D9 in the bleached samples (Figure 3d, Supporting Information Table S2). The defining intragenomic variant (DIV) counts for Symbiodiniaceae profiles show the breakdown of raw data in high resolution to predict taxa at each site and condition (Supporting Information Figure S2e–h). Principle Coordinate Analysis (PCoA) shows clear separation for the C3z/C3‐C3.10‐C3an, C3z‐C3‐C3.10‐C3bq and C15‐C15ad ITS2 type profiles along the axes where PCO1 explained 32.5% of the variation while Pco_2_ explained 25.1% (Figure 3e).

### 3.3 Bacterial community structure

Overall, the data set comprised 43 16S rRNA gene libraries (three replicates × four coral species × two sites × two coral conditions) totaling 1,412,100 sequences with a mean length of 294 bp. After quality filtering and exclusion of chimeras, 1,065,414 sequences were annotated to bacteria. Clustering of these sequences at the 97% similarity level resulted in 2,362 OTUs (Supporting Information Table S7), presented as a taxonomy stacked column plot to the phylogenetic level of family (Supporting Information Figure S3). Significant interactions in bacterial diversity were found between species, site, and colony condition for the number of OTUs per sample, phylogenetic diversity and Chao1 (Univariate GLM; see Table 1). There was a significant difference between coral species for the number of OTUs (One‐way ANOVA; *F*_3,42_ = 21.91, *p* < 0.001), phylogenetic diversity (*F*_3,42_ = 22.16, *p* < 0.001), Chao1 (*F*_3,42_ = 23.76, *p* < 0.001), Simpson's diversity (*F*_3,42_ = 6.70, *p* < 0.001), and Shannon's diversity (*F*_3,42_ = 14.74, *p* < 0.001). Also, we found a significant difference in Chao1 between sites (*F*_1,42_ = 22.26, *p* < 0.0001), but no differences detected for site or coral condition for the remaining parameters. Highest number of OTUs per sample was observed for *P. lutea* across all sites (average 208.46 ± 5.8), but with the highest number recorded for the bleached *C. aspera* samples from Praslin (360 ± 27) and lowest for the unbleached *A. gemmifera* samples at East Bay (55 ± 23; Table 1). Total species richness was generally highest for *C. aspera* with the maximum recorded for unbleached samples at East Bay (Chao1 = 662.96 ± 6.05) indicating a more diverse and heterogeneous bacterial community, and lowest for the bleached *A. muricata* samples from East Bay (60.93 ± 31.90; Table 1). Species evenness (Simpson's diversity) was highest for *P. lutea* ranging between 0.81 ± 0.16 and 0.97 ± 0.01, and lowest for *A. muricata* (ranging 0.41 ± 0.13 to 0.60 ± 0.09; Table 1). Bacterial diversity was highest for *P. lutea*, where Shannon's diversity values ranged from 4.58 ± 1.64 to 6.36 ± 0.21, and lowest for *A. muricata* (Shannon's diversity between 1.35 ± 0.39 and 2.71 ± 0.55; Table 1).

**Table 1:** Summary statistics for alpha diversity indices of 16S rRNA gene amplicon sequences for microbial communities associated with *Acropora gemmifera, Acropora muricata, Coelastrea aspera* and *Porites lutea* at East Bay and Praslin for each category of coral condition (i.e., bleached and unbleached)

Site	Condition	*n*	# reads	OTU/Sample	PD_whole_tree	Chao1	Simpsons	Shannons
*A. gemmifera*								
East Bay	Bleached	3	16,970.67 ± 848.99	67.33 ± 18.90	4.59 ± 0.99	130.76 ± 59.35	0.64 ± 0.14	2.73 ± 0.67
	Unbleached	3	14,076.33 ± 3,515.56	55.67 ± 23.80	4.16 ± 1.46	85.70 ± 22.37	0.65 ± 0.16	2.75 ± 1.00
Praslin	Bleached	3	19,063.67 ± 4,324.15	83.33 ± 14.97	5.48 ± 0.65	154.16 ± 28.44	0.78 ± 0.09	3.57 ± 0.66
	Unbleached	3	14,725.33 ± 2044.84	105.33 ± 18.76	6.67 ± 0.95	168.70 ± 34.66	0.82 ± 0.04	3.86 ± 0.49
*A. muricata*								
East Bay	Bleached	3	22,968.33 ± 4,380.83	30.00 ± 12.50	2.46 ± 0.84	60.93 ± 31.90	0.41 ± 0.13	1.35 ± 0.39
	Unbleached	3	15,612.67 ± 2,796.26	71.00 ± 23.71	5.03 ± 1.40	109.05 ± 26.55	0.60 ± 0.06	2.71 ± 0.55
Praslin	Bleached	3	16,103.67 ± 3,189.49	40.00 ± 8.88	3.08 ± 0.57	75.38 ± 16.22	0.60 ± 0.09	2.16 ± 0.43
	Unbleached	3	10,968.00 ± 1954.59	41.00 ± 13.01	3.06 ± 0.89	75.17 ± 33.10	0.64 ± 0.12	2.33 ± 0.63
*C. aspera*								
East Bay	Bleached	2	13,306.50 ± 5,128.50	184.50 ± 106.50	9.62 ± 3.95	306.88 ± 162.88	0.70 ± 0.28	4.61 ± 2.61
	Unbleached	2	12,907.00 ± 6,179.00	360.50 ± 27.50	15.80 ± 1.33	662.96 ± 6.05	0.99 ± 0.01	7.71 ± 0.24
Praslin	Bleached	3	16,738.33 ± 696.74	253.33 ± 23.56	12.29 ± 0.99	510.21 ± 40.09	0.93 ± 0.03	6.01 ± 0.35
	Unbleached	1	13,853.00 (n/a)	99.00 (n/a)	7.37 (n/a)	251.50 (n/a)	0.75 (n/a)	3.44 (n/a)
*P. lutea*								
East Bay	Bleached	3	24,632.67 ± 113.81	183.00 ± 49.21	10.27 ± 2.26	317.75 ± 90.47	0.94 ± 0.02	5.70 ± 0.54
	Unbleached	3	19,577.00 ± 6,361.46	277.67 ± 41.59	13.58 ± 1.65	470.00 ± 60.58	0.92 ± 0.05	6.24 ± 0.95
Praslin	Bleached	3	32,955.67 ± 5,782.41	141.67 ± 64.01	8.19 ± 2.96	269.59 ± 105.85	0.81 ± 0.16	4.58 ± 1.64
	Unbleached	2	25,426.50 ± 8,188.50	231.50 ± 65.50	12.58 ± 2.51	438.13 ± 145.99	0.97 ± 0.005	6.36 ± 0.21
*F*(*df*) statistic			*F*_3,42_ = 0.58	*F*_3,42_ = 3.76	*F*_3,42_ = 2.92	*F*_3,42_ = 4.10	*F*_3,42_ = 1.41	*F*_3,42_ = 1.87
*p* value			0.634	0.020*	0.049*	0.014*	0.259	0.152

Note

Statistical analysis (Univariate general linear model [GLM] for interactions between species ×site × coral condition are shown and significance is shown with an asterisk (*). Average ± *SE* are shown. n/a = no SE calculated for *C. aspera* unbleached sample from Praslin (*n* = 1).

Although there was no significant interaction for bacterial community composition between species, site, and coral condition, a significant difference was detected between species (PERMANOVA; pseudo *F*_3,47_ = 4.84, *p* < 0.0001, Supporting Information Table S3a). This was further confirmed with ANOSIM (Global R = 0.376, *p* < 0.0001; Supporting Information Table S3b) with significant differences between all species except *C. aspera* and *P. lutea*. No differences (Global R = −0.012, *p* = 0.568) were detected between bleached and unbleached coral samples, and once the most abundant (top three ranked by relative abundance) families were excluded from analysis (Hahellaceae, Rhodospirillaceae, and Rhodobacteraceae), an interaction between species (PERMANOVA; pseudo *F*_3,47_ = 5.20, *p* < 0.001; Supporting Information Table S4a) and species × site (pseudo *F*_3,47_ = 1.63, *p* < 0.020; Supporting Information Table S4a) became apparent. The largest difference detected was between *A. muricata* and *P. lutea* (ANOSIM; R statistic = 0.675, *p* = 0.0002; Supporting Information Table S4b) while the most similar species were *C. aspera* and *A. muricata* (ANOSIM; R statistic = 0.07, *p* = 0.102; Supporting Information Table S4b) when the most abundant families were excluded from analysis. This was not detected in the full dataset, highlighting the importance of additional analyses excluding the most abundant taxa to detect differences in the less abundant ones that can be masked by those in higher abundance.

**Figure 4:**
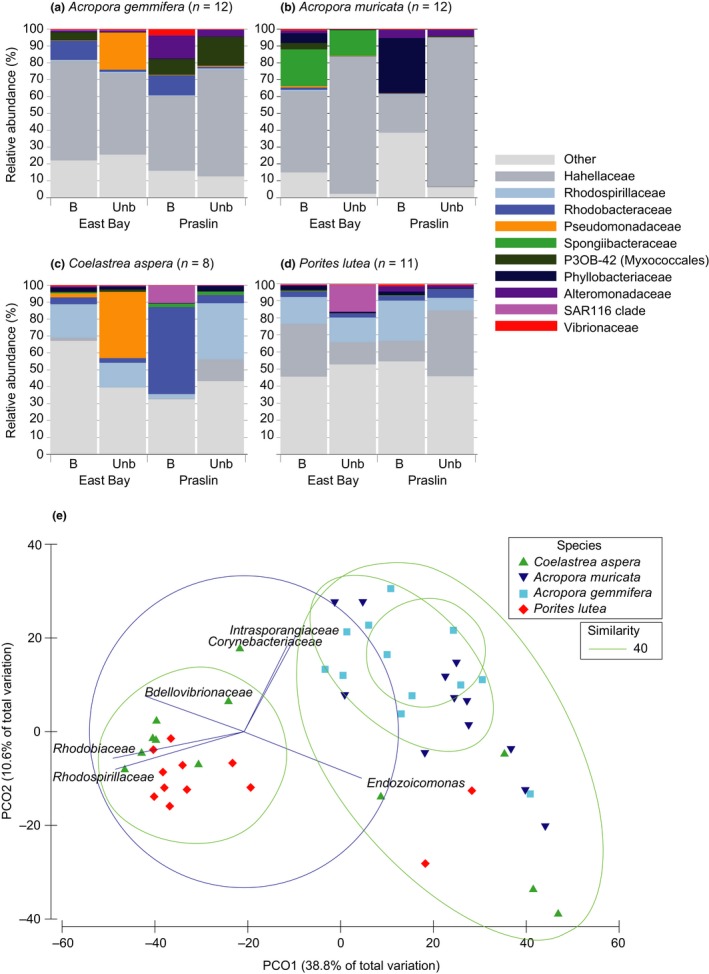
Average relative abundance (%) of bacterial community composition for (a) *Acropora gemmifera,* (b) *Acropora muricata*, (c) *Coelastrea aspera* and (d) *Porites lutea* classified as bleached or unbleached from East Bay and Praslin in Seychelles as a taxonomy stacked column plot to family level. Remaining taxa are grouped as “other”. Values displayed are mean relative abundances (*n* = 2–3). Each color represents one of the 10 most abundant bacterial families. (e) Principle Coordinate Analysis (PCoA) for the dominant bacterial taxa found in four coral species at East Bay and Praslin in Seychelles. Data was fourth‐root transformed, and a Bray‐Curtis similarity matrix was used with a correlation of 0.2. Ellipses denote similarity clusters of 20% (green dashed line). Percentages on axes indicate variation explained by the two coordinates

Similarity percentage analysis was used to identify the main contributing bacterial families within each of the coral species. Hahellaceae were the top contributing bacterial family in all coral species, contributing 31.25% in *A. gemmifera*, 44.87% in *A. muricata*, 15.78% in *C. aspera,* and 10.45% in *P. lutea* (Figure 4a–d, Supporting Information Table S5). Alteromonadaceae were the second highest contributing bacterial family for both *Acropora* sp. (4.81% *A. gemmifera* and 5.05% in *A. muricata*) while Rhodospirillaceae and Rhodobacteraceae comprised the top 3 contributing taxa for *C. aspera* and *P. lutea* (Supporting Information Table S5). PCoA of the dominant bacterial communities showed a distinct community composition for *A. gemmifera* and *A. muricata*, clustering separately from the majority of *P. lutea* and *C. aspera* (Figure 4e) along the primary axis (PCO1), explaining 38.8% of the total variation.

## 4 DISCUSSION

Microbiomes play a key role in contributing to coral fitness over space and time (Putnam et al., 2017; Suggett et al., 2017) and are known to exhibit broad changes across reefs persisting under different environmental conditions (Roder et al., 2015) and when subjected to atypical stress (Grottoli et al., 2018; Röthig et al., 2016; Ziegler, Seneca et al., 2017b). Here, we provide the first characterization of the microbial community composition (i.e., Symbiodiniaceae and bacteria) for key reef‐building coral taxa of Seychelles across two different environments. In addition, we characterize the microbial communities associated with states of coral health (i.e., bleached and unbleached), collected during the 2016 marine heat wave that induced mass coral bleaching and mortality. In examining coral species that have previously been shown to be broadly heat stress sensitive (*A. muricata*,* A. gemmifera*) and tolerant (*P. lutea*,* C. aspera*) in Seychelles (Harris et al., 2014) and WIO (McClanahan et al., 2014, 2007 ), we have shown that bleaching susceptibility among coral species is indeed broadly consistent with differences in microbiomes. However, conserved microbiome signatures observed for bleached and unbleached colonies of all coral species suggest complex regulation of bleaching severity by the coral holobiont and genotype.

### 4.1 Decline in coral cover during 2016 mass bleaching

Heat stress is recognized as the most common cause of coral bleaching, and high record temperatures between 2015 and 2017 triggered the third global mass bleaching event, the most damaging to date (Hughes et al., 2018, 2017 ). At CMNP, about 40% of all corals bleached in April 2016 (Figure 2a), in particular species of *Acropora* and *Pocillopora* that resulted in large declines in coral cover recorded in April 2017. Specifically, decline of *Acropora* sp. and *Pocillopora* sp. from ca. 15%–20% (2016) to 1%–2% of total benthic cover was recorded (2017; Figure 2c). Such dramatic loss of these same taxa was similarly observed during previous recent marine heat wave events in Seychelles (Graham et al., 2008; Wilson et al., 2012) and other sites in the WIO (Baker, McClanahan, Starger, & Boonstra, 2013; McClanahan et al., 2014, 2007 ), reflecting the typically stress‐sensitive “boom and bust” nature of commonly fast‐growing branching taxa (Darling, Alvarez‐Filip, Oliver, McClanahan, & Côté, 2012; Zinke et al., 2018). The significant decline in abundance of *Acropora* sp. and *Pocillopora* sp. in 2016 suggests that these populations in Seychelles remain inherently susceptible to heat stress, and currently show no evidence for acclimatization to recent repeat heat wave events in this region unlike other WIO reef locations (Kenya; McClanahan, 2017).

### 4.2 Species‐specific Symbiodiniaceae composition

As expected, the major symbiont communities for corals sampled in CMNP broadly reflected those sampled from other Indo‐Pacific regions. For example, ITS2 type C3 (and additional types characterized by sequences from the C3 radiation; Thornhill, Lewis, Wham, & LaJeunesse, 2014) for *Acropora* sp. in Eastern Africa (Chauka, 2012), the Chagos Archipelago (Yang et al., 2012), Red Sea (Ziegler, Eguíluz et al., 2017a) and Persian‐Arabian Gulf (Hume et al., 2013, 2016; Smith, Ketchum et al., 2017; Smith, Vaughan et al., 2017). The C3 sequence represents one of several major radiations within the genus *Cladocopium* (Thornhill et al., 2014) and our C3z type profile among *Acropora* sp. sampled here is 2 bp different from the C3 basal sequence. As such, it is currently unclear how genetically and/or phenotypically comparable our C3 type(s) are to these reports from elsewhere in the WIO previously (but see Ziegler, Eguíluz et al., 2017a for the Red Sea). Heat stress tolerance is clearly highly variable among the C3 radiation (Hume et al., 2013, 2015 ). However, the mass bleaching response for *Acropora* sp. within the CMNP commonly hosting C3z‐C3 would suggest that this *Cladocopium* type is inherently heat stress sensitive, and a highly conserved *Acropora* sp. host‐symbiont association. While we did not assess symbiont types associated with *Pocillopora* sp. that also experienced mass bleaching in CMNP, previous observations from the Chagos Archipelago (Yang et al., 2012) and Tanzania (Chauka, 2012) have identified almost synonymous associations with the C1 group (*Pocillopora damicornis*,* Pocillopora verrucosa*,* Pocillopora eydouxi*), another major radiation within *Cladocopium* (Thornhill et al., 2014).

Alternate host‐symbiont associations were observed among the coral taxa that exhibited comparatively little mass bleaching within CMNP, *P. lutea* and *C. aspera*. As with elsewhere in Eastern Africa (Chauka, 2012), the Red Sea and Persian‐Arabian Gulf (Smith, Ketchum et al., 2017; Smith, Vaughan et al., 2017; Ziegler, Eguíluz et al., 2017a) and Pacific (e.g., LaJeunesse et al., 2004; LaJeunesse et al., 2003), *P. lutea* in the CMNP almost exclusively associated with ITS2 type C15. Perhaps most intriguingly was the high diversity of Symbiodiniaceae in *C. aspera* (formerly known as *Goniastrea aspera*—see Huang et al., 2014) comprising predominantly D1–D4 (–D6) ITS2 type profiles, in comparison to previously being associated with C3 types in the Indian Ocean (LaJeunesse et al., 2010), C1 types in Western Australia (Silverstein, Correa, LaJeunesse, & Baker, 2011), and D1a types in Thailand (Brown, Dunne, Edwards, Sweet, & Phongsuwan, 2015). Thermally tolerant *D. trenchii* (ITS2 type profile D1–4 is routinely observed in the WIO with a wide host range and where more than one Symbiodiniaceae type is detected within a coral species (LaJeunesse et al., 2010; see alsoSmith, Ketchum et al., 2017; Smith, Vaughan et al., 2017).

Bleaching observed for the heat stress‐sensitive taxa, *A. muricata* and *A. gemmifera*, in both reef environments was reflected by a loss of symbiont density (Figure 2e–h) and shifts in dominant C3 radiation types (Figure 3a,b; Supporting Information Figure S2), whereas the few unbleached colonies sampled at Praslin contained D1 types (*A. muricata*) or C3z/C3 types (*A. gemmifera*). Similarly, bleached colonies of the heat stress‐tolerant taxa were characterized by a loss of Symbiodiniaceae cells (but maintaining type C15 dominance, *P. lutea*) or alternate symbiont types (changing from D1 to predominantly C3 types, *C. aspera*). Together, these highlight important localized regulatory processes of bleaching susceptibility within these reefs. First, given the apparent stress‐sensitive nature of our *Acropora* sp. C3z/C3 associations, lack of bleaching for *A. gemmifera* at Praslin may indicate small‐scale environmental variability, as similarly observed for in‐shore *Acropora sp*. colonies on the Great Barrier Reef also in 2016 (Hoogenboom et al., 2017). Turbidity has been suggested to be critical in providing refuge against bleaching intensity (LaJeunesse et al., 2010; Oliver & Palumbi, 2009; Ulstrup & Van Oppen 2003). However, the mass bleaching observed at both our relatively turbid and clear sites (of similar extent, Figure 2) would thus suggest small‐scale environmental variability afforded through complex reef habitats (e.g., shading from overhangs, Cacciapaglia & Woesik, 2016) are more important in providing refuge from heat stress. Bleaching of *P. lutea* and loss of C15 type cells at both sites similarly suggests localized small‐scale amplification of heat stress, for example, by high light (Hoogenboom et al., 2017). Second, alternate symbiont types of unbleached typically heat‐sensitive *A. muricata* (D1 type, Praslin) and bleached typically heat‐tolerant *C. aspera* (D1 type) reflects some capacity for Symbiodiniaceae community re‐organization needed for thermal acclimatization. *D. trenchii* (ITS2 type profile D1–D4) is a known coral host‐generalist (LaJeunesse et al., 2010, 2014) and heat stress‐tolerant taxon (LaJeunesse, Smith, Finney, & Oxenford, 2009). While it is unclear why unbleached *A. muricata* only associated with D1 types at Praslin, enhanced prevalence of *Durusdinium* spp. is consistent with increased turbidity (LaJeunesse et al., 2010; Oliver & Palumbi, 2009) that is typical of Praslin.

### 4.3 Stability in bacterial communities under thermal stress

Differences in bleaching susceptibility among taxa that are not easily resolvable through Symbiodiniaceae identity or host phenotype alone may result from differences in the associated bacterial communities (Morrow et al., 2018; Sweet & Bulling, 2017). Given the putative critical contribution of bacteria to coral holobiont functioning, changes in the identity and abundance of associated bacteria may allow for rapid acclimatization or adaptation to environmental change (Bourne et al., 2008; Cárdenas et al., 2012; Roder et al., 2013; Roder, Arif, Daniels, Weil, & Voolstra, 2014; Vega Thurber et al., 2009). Community shifts (such as a decrease in *Endozoicomonas* sp.) can occur in visibly healthy corals in degraded ecosystems (Ziegler et al., 2016) and changes to the bacterial community prior to visual signs bleaching (e.g., increase in *Vibrio* related sequences) have been shown to occur (Bourne et al., 2008). In contrast, we did not observe such obvious bacterial community shifts between bleached and unbleached conspecifics for heat stress susceptible or resistant coral species in Seychelles. This outcome is surprising since bacterial communities are expected to exhibit more rapid responses to stressors than *Symbiodinium* due to faster metabolism and generation times (Pogoreutz et al., 2018). As such, the observed bleaching susceptibility in our study may be more likely driven by differences in Symbiodiniaceae (via stress susceptibility) rather than by the hosts’ bacterial assemblages. That said, we acknowledge that our opportunistic sampling during the 2016 mass bleaching event meant that we were not able to compare samples before and after the beaching event. While more resolute temporal sampling throughout bleaching is needed to resolve for potentially different dynamics (and the role) of Symbiodiniaceae versus bacterial communities during heat stress, the direct comparison of bleached and unbleached colonies for all coral taxa during the heat stress allowed us to uniquely assess a putative contribution of bacterial taxa to bleaching susceptibility.

We identified three dominant bacterial families across all factors (species, site, and coral condition), suggesting a putatively important potential role in coral health and function due to their widespread prevalence and high abundance. Bacterial community stability was largely attributed to the abundance of Hahellaceae (comprising *Endozoicomonas*—ranging between 10% and 44% for all coral species; Supporting Information Table S5), congruent with recent work (Bayer et al., 2013; Neave, Apprill, Ferrier‐Pagès, & Voolstra, 2016; Neave, Rachmawati, et al., 2017a; Pogoreutz et al., 2018; Pootakham et al., 2017). While the function of *Endozoicomonas* has not yet been defined, their genomes are significantly enriched in genes for carbohydrate transport and recycling as well as for protein and amino acid provision (Neave, Michell, Apprill, & Voolstra, 2017b) and phenotypic assays confirm a high metabolic versatility in vitro (Yang et al., 2010). Despite the high abundance of *Endozoicomonas* in apparently healthy corals (Apprill, Hughen, & Mincer, 2013; Roder et al., 2015), strongly reduced abundances have been reported for stressed, diseased or bleached corals, suggesting they may be an indicator of coral health or habitat suitability (Bourne et al., 2008; Cárdenas et al., 2012; Meyer, Paul, & Teplitski, 2014; Röthig et al., 2016; Ziegler et al., 2016). However, similar to previous findings in bleached Red Sea *P*. *verrucosa* (Pogoreutz et al., 2017), we did not observe reduced abundances of *Endozoicomonas* in bleached colonies, even though colonies were visibly strongly bleached 1 week prior to sampling (see methods).

Alteromonadales and Rhodobacteraceae are commonly identified as members of the coral microbiome including larvae and juvenile early stage colonies (Apprill, Weber, & Santoro, 2016), some of which are able to degrade and assimilate dimethylsulfoniopropionate (DMSP; Reisch, Moran, & Whitman, 2011), an abundant carbon source in corals (Raina et al., 2013). Members of both bacterial families have previously been observed in visibly healthy and stressed corals (Li et al., 2014; Pantos, Bongaerts, Dennis, Tyson, & Hoegh‐Guldberg, 2015) and were fairly abundant in some of the investigated coral species in the present study (Alteromonadaceae were 3%–4% relative abundance and Rhodobacteraceae were up to 5% relative abundance; for details refer to Supporting Information Table S5). Given their presence in a range of hosts and environments (including seawater), it suggests this taxon is metabolically flexible and may provide important functions to the coral holobiont (Röthig et al., 2016).

Vibrionaceae are an opportunistic and potentially pathogenic bacterial family commonly associated with coral disease and have previously been linked to bleaching (Bourne et al., 2008; Garren et al., 2015; Tout et al., 2015). We found Vibrionaceae in all corals, albeit in lower abundance, ranging between a contribution of 1.12% (*P. lutea*) to 2.61% (*A. muricata*). The reported association of Vibrionaceae associated with unimpaired, apparently healthy corals has been reported (Bourne & Munn, 2005). Nevertheless, the consistent association of these bacteria suggests that corals in Seychelles harbor stable, and presumably locally adjusted microbiomes, allowing the corals to cope well in the ambient environment (sensu Hernandez‐Agreda, Leggat, Bongaerts, & Ainsworth, 2016).

### 4.4 Bacterial diversity aligns with bleaching susceptibility

Healthy corals generally comprise specific, stable, and uneven microbial assemblages indicating host‐selected microbiomes (Bayer et al., 2013; Bourne et al., 2008). We observed the highest species richness (Chao1), evenness (Simpson's), and bacterial diversity (Shannon's) in the more heat‐tolerant massive corals (*P. lutea*, followed by *C. aspera*) compared with stress‐sensitive branching corals (*A. muricata* higher than *A. gemmifera*), consistent with findings from Liang et al. (2017). *Acropora muricata* also exhibited a highly uneven bacterial community that was dominated by *Endozoicomonas* (up to 44% contribution), compared with *P. lutea* that had the highest evenness and diversity, with only a 10% contribution of *Endozoicomonas* (Table 1, Supporting Information Table S5). The higher bacterial diversity found at Praslin (the turbid site) compared to East Bay (clear water site) is also supported by work showing corals on anthropogenically impacted reefs harbor higher bacterial diversity than those further from the disturbance (Morrow, Moss, Chadwick, & Liles, 2012). Praslin is within a bay bordered by human populations and thus more prone to anthropogenic influence from run‐off and pollution, compared with East Bay (which is located further from human influence off Curieuse Island). Furthermore, bacterial community composition in corals from more impacted shallow sites can display higher intraspecies variability with thermal stress (Littman, Willis, & Bourne, 2011). Consequently, it is plausible that the changes in bacterial communities we observed between sites may in part reflect differences in overall reef health through proximity to more localized human populations at Praslin compared to East Bay.

Responses of less abundant or rare members of the bacterial microbiome may not become apparent until the dominant bacterial members are excluded from analysis. Indeed, we identified a significant interaction between species and site (Supporting Information Table S4) when the most abundant families were excluded from analysis. Here, the bacterial family contributing the highest percentage for both sites was Enterobacteriaceae, with Vibrionaceae also in the top three. In contrast, East Bay had a 2.94% contribution from Pseudomonadaceae while Praslin had Alteromonadaceae (7.7%) in the top three dominant taxa (once the most abundant families were excluded). This difference was only detected upon exclusion of the most abundant families, and a comparison of bacterial taxa for coral condition revealed no difference in the order of the most abundant taxa (Enterobacteriaceae, Alteromonadaceae and Vibrionaceae for unbleached and bleached). Such site‐specific differences in dominant bacterial taxa have similarly been reported for the heat‐sensitive species (*Acropora* sp.) exposed to a range of stressors (Apprill et al., 2016; Littman, Willis, Pfeffer, & Bourne, 2009; McDevitt‐Irwin, Baum, Garren, & Vega Thurber, 2017; Pantos et al., 2015). As such, the low flexibility of bacterial communities associated with heat‐sensitive corals in Seychelles may reduce their ability to rapidly respond to environmental stress (Pogoreutz et al., 2018), as highlighted by the significant loss in coral cover reported during the 2016–2017 mass bleaching event for *Acropora* sp. (and *Pocillopora* sp.; Figure 1a).

Finally, it is possible that suboptimal partnerships with Symbiodiniaceae algal symbionts could enhance vulnerability to opportunistic bacterial infection. As such, changing Symbiodiniaceae taxa following bleaching events may not only provide immediate benefits to the coral holobiont in terms of thermal tolerance, but could also result in a longer‐term trade‐off with disease resistance (Littman, Bourne, & Willis, 2010). Recent work has shown coral host intraspecific differences in Symbiodiniaceae composition correlated with disease susceptibility (Rouzé, Lecellier, Saulnier, & Berteaux‐Lecellier, 2016). Specifically, the predisposition to disease and infection by *Vibrio* spp. was positively correlated with Symbiodiniaceae genus *Symbiodinium* (formerly clade A), but negatively correlated with *Durusdinium* in *Acropora cytherea* (Rouzé et al., 2016). In our study, we found *Durusdinium* types D4 and D9 unique to *P. lutea* (and bleached samples of *C. aspera*) and previous work has shown increased occurrence of *Durusdinium* types is consistent with increased turbidity. Furthermore, *Durusdinium*‐infected corals have shown shifts in the associated bacterial community under heat stress, while no shifts were reported for *Cladocopium* within the same coral host (Littman et al., 2010). It might therefore be informative to examine for bacterial associates that co‐occur with Symbiodiniaceae types in future studies.

In characterizing the microbiome composition of four species of Seychelles’ corals during the most severe mass bleaching event on record, we have shown that susceptibility to stressors is reflected by underlying microbiome community structures. Specifically, bleaching susceptibility among coral taxa corresponds largely to differences in specific host‐Symbiodiniaceae associations, while the bacterial microbiome community remains largely stable. As such, unbleached colonies of bleaching‐susceptible corals likely persist through availability of small‐scale (“micro”) environmental refuges that can dampen the effect of heat stress, (e.g., shading from surrounding substrate). While bacterial communities were highly similar between bleached and unbleached corals, differences were observed between species, adding to previous evidence for species‐specificity. Thus, microbiome profiling of both Symbiodiniaceae and bacterial communities may provide new capacity to more broadly identify stress susceptible versus tolerant coral populations, which is needed to aid targeted management in reef systems such as Seychelles.

## CONFLICT OF INTEREST

None declared.

## AUTHOR CONTRIBUTION

EFC, DJSm, and DJSu designed and conceived the experiment. BCCH, CP, and CRV contributed new reagents or analytical tools. SGG, EFC, DJSm, TK, EOO, GG, BCCH, CP, CRV, and DJSu performed research. All authors analyzed and interpreted data. SGG, DJSu and EFC wrote the first draft of the manuscript. All authors contributed to and approved the manuscript.

## Data Availability

Raw sequencing data determined in this study are available under NCBI BioProject ID PRJNA477731 (https://www.ncbi.nlm.nih.gov/bioproject/PRJNA477731) for 16S rRNA gene data and under NCBI BioProject ID PRJNA477732 (https://www.ncbi.nlm.nih.gov/bioproject/PRJNA477732) for ITS2 sequence data. Other data are available in the Supporting Information.

## References

[ece34662-bib-0001] Anderson, M. J. , Gorley, R. N. , & Clarke, K. R. (2008). PERMANOVA+ for PRIMER: Guide to software and statistical methods. Plymouth, UK: PRIMER‐E Ltd.

[ece34662-bib-0002] Andersson, A. F. , Lindberg, M. , Jakobsson, H. , Bäckhed, F. , Nyrén, P. , & Engstrand, L. (2008). Comparative analysis of human gut microbiota by barcoded pyrosequencing. PLoS ONE, 3, e2836 10.1371/journal.pone.0002836. 18665274 PMC2475661

[ece34662-bib-0003] Apprill, A. , Hughen, K. , & Mincer, T. (2013). Major similarities in the bacterial communities associated with lesioned and healthy Fungiidae corals. Environmental Microbiology, 15, 2063–2072. 10.1111/1462-2920.12107. 23516962

[ece34662-bib-0004] Apprill, A. , Weber, L. G. , & Santoro, A. E. (2016). Distinguishing between microbial habitats unravels ecological complexity in coral microbiomes. mSystems, 1(5), pii: e00143‐16 10.1128/mSystems.00143-16. PMC5080407 27822559

[ece34662-bib-0005] Arif, C. , Daniels, C. , Bayer, T. , Banguera‐Hinestroza, E. , Barbrook, A. , Howe, C. J. , … Voolstra, C. R. (2014). Assessing symbiodinium diversity in scleractinian corals via next‐generation sequencing‐based genotyping of the ITS2 rDNA region. Molecular Ecology, 23, 4418–4433. 25052021 10.1111/mec.12869 PMC4285332

[ece34662-bib-0006] Baker, A. C. , McClanahan, T. R. , Starger, C. J. , & Boonstra, R. K. (2013). Long‐term monitoring of algal symbiont communities in corals reveals stability is taxon dependent and driven by site‐specific thermal regime. Marine Ecology Progress Series, 479, 85–97. 10.3354/meps10102.

[ece34662-bib-0007] Bayer, T. , Neave, M. J. , Alsheikh‐Hussain, A. , Aranda, M. , Yum, L. K. , Mincer, T. , … Voolstra, C. R. (2013). The microbiome of the Red Sea coral *Stylophora pistillata* is dominated by tissue‐associated Endozoicomonas bacteria. Applied and Environmental Microbiology, 79, 4759–4762. 10.1128/AEM.00695-13. 23709513 PMC3719505

[ece34662-bib-0008] Bourne, D. , Iida, Y. , Uthicke, S. , & Smith‐Keune, C. (2008). Changes in coral‐associated microbial communities during a bleaching event. ISME Journal, 2, 350–363. 10.1038/ismej.2007.112. 18059490

[ece34662-bib-0009] Bourne, D. G. , & Munn, C. B. (2005). Diversity of bacteria associated with the coral *Pocillopora damicornis* from the Great Barrier Reef. Environmental Microbiology, 7, 1162–1174. 10.1111/j.1462-2920.2005.00793.x. 16011753

[ece34662-bib-0010] Brown, B. E. , Dunne, R. P. , Edwards, A. J. , Sweet, M. J. , & Phongsuwan, N. (2015). Decadal environmental ‘memory’ in a reef coral? Marine Biology, 162, 479–483. 10.1007/s00227-014-2596-2.

[ece34662-bib-0011] Cacciapaglia, C. , & Woesik, R. (2016). Climate‐change refugia: Shading reef corals by turbidity. Global Change Biology, 22, 1145–1154. 10.1111/gcb.13166. 26695523

[ece34662-bib-0012] Camacho, C. , Coulouris, G. , Avagyan, V. , Ma, N. , Papadopoulos, J. , Bealer, K. , & Madden, T. L. (2009). BLAST+: Architecture and applications. BMC Bioinformatics, 10, 421 10.1186/1471-2105-10-421. 20003500 PMC2803857

[ece34662-bib-0013] Camp, E. F. , Smith, D. J. , Evenhuis, C. , Enochs, I. , Manzello, D. , Woodcock, S. , & Suggett, D. J. (2016). Acclimatization to high‐variance habitats does not enhance physiological tolerance of two key Caribbean corals to future temperature and pH. Proceedings of the Royal Society B: Biological Sciences, 283(1831), 20160442 10.1098/rspb.2016.0442. PMC4892798 27194698

[ece34662-bib-0014] Caporaso, J. G. , Kuczynski, J. , Stombaugh, J. , Bittinger, K. , Bushman, F. D. , Costello, E. K. , … Knight, R. (2010). QIIME allows analysis of high‐throughput community sequencing data. Nature Methods, 7, 335–336. 10.1038/nmeth.f.303. 20383131 PMC3156573

[ece34662-bib-0015] Cárdenas, A. , Rodriguez‐R, L. M. , Pizarro, V. , Cadavid, L. F. , & Arévalo‐Ferro, C. (2012). Shifts in bacterial communities of two caribbean reef‐building coral species affected by white plague disease. The ISME Journal, 6, 502–512. 10.1038/ismej.2011.123. 21955993 PMC3280130

[ece34662-bib-0016] Casey, K. S. , Selig, E. R. , Zhang, D. , Saha, K. , Krishnan, A. , & McMichael, E. (2015). The coral reef temperature anomaly database (CoRTAD) version 5 ‐ Global, 4 km sea surface temperature and related thermal stress metrics for 1982–2012 (NCEI Accession 0126774). Version 1.1. NOAA National Centers for Environmental Information. Dataset. doi:10.7289/V5CZ3545

[ece34662-bib-0017] Chauka, L. J. (2012). Diversity of the symbiotic alga Symbiodinium in Tanzanian scleractinian coral. Western Indian Ocean Journal of Marine Science, 11, 67–76.

[ece34662-bib-0018] Chong‐Seng, K. M. , Graham, N. A. J. , & Pratchett, M. S. (2014). Bottlenecks to coral recovery in the Seychelles. Coral Reefs, 33, 449–461. 10.1007/s00338-014-1137-2.

[ece34662-bib-0019] Clarke, K. R. , & Gorley, R. N. (2006). PRIMER v6: User manual/tutorial. Plymouth, UK: PRIMER‐E.

[ece34662-bib-0020] Clifton, J. , Etienne, M. , Barnes, D. K. A. , Barnes, R. S. K. , Suggett, D. J. , & Smith, D. J. (2012). Marine conservation policy in Seychelles: Current constraints and prospects for improvement. Marine Policy, 36, 823–831. 10.1016/j.marpol.2011.11.009.

[ece34662-bib-0021] Coleman, A. W. , Suarez, A. , & Goff, L. J. (1994). Molecular delineation of species and syngens in volvocacean green algae (chlorophyta). Journal of Phycology, 30, 80–90.

[ece34662-bib-0022] Darling, E. S. , Alvarez‐Filip, L. , Oliver, T. A. , McClanahan, T. R. , & Côté, I. M. (2012). Evaluating life‐history strategies of reef corals from species traits. Ecology Letters, 15, 1378–1386. 10.1111/j.1461-0248.2012.01861.x. 22938190

[ece34662-bib-0023] Eren, A. M. , Morrison, H. G. , Lescault, P. J. , Reveillaud, J. , Vineis, J. H. , & Sogin, M. L. (2015). Minimum entropy decomposition: Unsupervised oligotyping for sensitive partitioning of high‐throughput marker gene sequences. The ISME Journal, 9, 968 10.1038/ismej.2014.195. 25325381 PMC4817710

[ece34662-bib-0024] Frias‐Lopez, J. , Zerkle, A. L. , Bonheyo, G. T. , & Fouke, B. W. (2002). Partitioning of bacterial communities between seawater and healthy, black band diseased, and dead coral surfaces. Applied and Environmental Microbiology, 68, 2214–2228. 10.1128/AEM.68.5.2214-2228.2002. 11976091 PMC127591

[ece34662-bib-0025] Garren, M. , Son, K. , Tout, J. , Seymour, J. R. , & Stocker, R. (2015). Temperature‐induced behavioral switches in a bacterial coral pathogen. The ISME Journal, 10, 1363 10.1038/ismej.2015.216. 26636553 PMC5029190

[ece34662-bib-0026] Glasl, B. , Herndl, G. J. , & Frade, P. R. (2016). The microbiome of coral surface mucus has a key role in mediating holobiont health and survival upon disturbance. The ISME Journal, 10, 2280–2292. 10.1038/ismej.2016.9. 26953605 PMC4989324

[ece34662-bib-0027] Graham, N. A. J. , Jennings, S. , MacNeil, M. A. , Mouillot, D. , & Wilson, S. K. (2015). Predicting climate‐driven regime shifts versus rebound potential in coral reefs. Nature, 518, 94–97. 10.1038/nature14140. 25607371

[ece34662-bib-0028] Graham, N. A. J. , McClanahan, T. R. , MacNeil, M. A. , Wilson, S. K. , Polunin, N. V. C. , Jennings, S. , … Sheppard, C. R. C. (2008). Climate warming, marine protected areas and the ocean‐scale integrity of coral reef ecosystems. PLoS ONE, 3, e3039 10.1371/journal.pone.0003039. 18728776 PMC2516599

[ece34662-bib-0029] Graham, N. A. J. , Wilson, S. K. , Jennings, S. , Polunin, N. V. C. , Bijoux, J. P. , & Robinson, J. (2006). Dynamic fragility of oceanic coral reef ecosystems. Proceedings of the National Academy of Sciences of the United States of America, 103, 8425–8429. 10.1073/pnas.0600693103. 16709673 PMC1482508

[ece34662-bib-0030] Grottoli, A. G. , Dalcin Martins, P. , Wilkins, M. J. , Johnston, M. D. , Warner, M. E. , Cai, W.‐J. , … Schoepf, V. (2018). Coral physiology and microbiome dynamics under combined warming and ocean acidification. PLoS ONE, 13, e0191156 10.1371/journal.pone.0191156. 29338021 PMC5770069

[ece34662-bib-0031] Guest, J. R. , Low, J. , Tun, K. , Wilson, B. , Ng, C. , Raingeard, D. , … Steinberg, P. D. (2016). Coral community response to bleaching on a highly disturbed reef. Scientific Reports, 6, 20717 10.1038/srep20717. 26876092 PMC4753424

[ece34662-bib-0032] Hadaidi, G. , Rothig, T. , Yum, L. K. , Ziegler, M. , Arif, C. , Roder, C. , … Voolstra, C. R. (2017). Stable mucus‐associated bacterial communities in bleached and healthy corals of *Porites lobata* from the Arabian Seas. Scientific Reports, 7, 45362 10.1038/srep45362. 28361923 PMC5374439

[ece34662-bib-0033] Harris, A. , Wilson, S. , Graham, N. , & Sheppard, C. (2014). Scleractinian coral communities of the inner Seychelles 10 years after the 1998 mortality event. Aquatic Conservation: Marine and Freshwater Ecosystems, 24, 667–679. 10.1002/aqc.2464.

[ece34662-bib-0034] Hernandez‐Agreda, A. , Leggat, W. , Bongaerts, P. , & Ainsworth, T. D. (2016). The Microbial signature provides insight into the mechanistic basis of coral success across reef habitats. Mbio, 7, pii: e00560‐16 10.1128/mBio.00560-16. PMC4981706 27460792

[ece34662-bib-0035] Hoogenboom, M. O. , Frank, G. E. , Chase, T. J. , Jurriaans, S. , Álvarez‐Noriega, M. , Peterson, K. , … Paley, A. S. (2017). Environmental drivers of variation in bleaching severity of Acropora species during an extreme thermal anomaly. Frontiers in Marine Science, 4, 376 10.3389/fmars.2017.00376.

[ece34662-bib-0036] Howells, E. J. , Beltran, V. H. , Larsen, N. W. , Bay, L. K. , Willis, B. L. , & van Oppen, M. J. H. (2012). Coral thermal tolerance shaped by local adaptation of photosymbionts. Nature Climate Change, 2, 116–120. 10.1038/nclimate1330.

[ece34662-bib-0037] Huang, D. , Benzoni, F. , Fukami, H. , Knowlton, N. , Smith, N. D. , & Budd, A. F. (2014). Taxonomic classification of the reef coral families Merulinidae, Montastraeidae, and Diploastraeidae (Cnidaria: Anthozoa: Scleractinia). Zoological Journal of the Linnean Society, 171, 277–355. 10.1111/zoj.12140.

[ece34662-bib-0038] Hughes, T. P. , Anderson, K. D. , Connolly, S. R. , Heron, S. F. , Kerry, J. T. , Lough, J. M. , … Wilson, S. K. (2018). Spatial and temporal patterns of mass bleaching of corals in the Anthropocene. Science, 359, 80–83. 10.1126/science.aan8048. 29302011

[ece34662-bib-0039] Hughes, T. P. , Kerry, J. T. , Álvarez‐Noriega, M. , Álvarez‐Romero, J. G. , Anderson, K. D. , Baird, A. H. , … Wilson, S. K. (2017). Global warming and recurrent mass bleaching of corals. Nature, 543, 373–377. 10.1038/nature21707. 28300113

[ece34662-bib-0040] Hume, B. C. C. , D'Angelo, C. , Burt, J. , Baker, A. C. , Riegl, B. , & Wiedenmann, J. (2013). Corals from the Persian/Arabian Gulf as models for thermotolerant reef‐builders: Prevalence of clade C3 Symbiodinium, host fluorescence and ex situ temperature tolerance. Marine Pollution Bulletin, 72, 313–322. 10.1016/j.marpolbul.2012.11.032. 23352079

[ece34662-bib-0041] Hume, B. C. C. , D'Angelo, C. , Burt, J. A. , & Wiedenmann, J. (2018). Fine‐scale biogeographical boundary delineation and sub‐population resolution in the symbiodinium thermophilum coral symbiont group from the Persian/Arabian Gulf and Gulf of Oman. Frontiers in Marine Science, 5, 138 10.3389/fmars.2018.00138.

[ece34662-bib-0042] Hume, B. C. C. , D'Angelo, C. , Smith, E. G. , Stevens, J. R. , Burt, J. , & Wiedenmann, J. (2015). *Symbiodinium thermophilum* sp. nov., a thermotolerant symbiotic alga prevalent in corals of the world's hottest sea, the Persian/Arabian Gulf. Scientific Reports, 5, 8562 10.1038/srep08562. 25720577 PMC4342558

[ece34662-bib-0043] Hume, B. C. C. , Voolstra, C. R. , Arif, C. , D’Angelo, C. , Burt, J. A. , Eyal, G. , … Wiedenmann, J. (2016). Ancestral genetic diversity associated with the rapid spread of stress‐tolerant coral symbionts in response to Holocene climate change. Proceedings of the National Academy of Sciences of the United States of America, 113, 4416–4421. 10.1073/pnas.1601910113. 27044109 PMC4843444

[ece34662-bib-0044] Januchowski‐Hartley, F. A. , Graham, N. A. J. , Wilson, S. K. , Jennings, S. , & Perry, C. T. (2017). Drivers and predictions of coral reef carbonate budget trajectories. Proceedings of the Royal Society B: Biological Sciences, 284 10.1098/rspb.2016.2533. PMC5310043 28123092

[ece34662-bib-0045] Jennings, S. , Grandcourt, E. M. , & Polunin, N. V. C. (1995). The effects of fishing on the diversity, biomass and trophic structure of Seychelles’ reef fish communities. Coral Reefs, 14, 225–235. 10.1007/BF00334346.

[ece34662-bib-0046] Jessen, C. , Villa Lizcano, J. F. , Bayer, T. , Roder, C. , Aranda, M. , Wild, C. , & Voolstra, C. R. (2013). In‐situ effects of eutrophication and overfishing on physiology and bacterial diversity of the Red Sea Coral *Acropora hemprichii* . PLoS ONE, 8, e62091. 23630625 10.1371/journal.pone.0062091 PMC3632597

[ece34662-bib-0047] Jones, A. M. , Berkelmans, R. , van Oppen, M. J. H. , Mieog, J. C. , & Sinclair, W. (2008). A community change in the algal endosymbionts of a scleractinian coral following a natural bleaching event: Field evidence of acclimatization. Proceedings of the Royal Society of London B: Biological Sciences, 275 10.1098/rspb.2008.0069. PMC2367621 18348962

[ece34662-bib-0048] Kahlke, T. (2018). Ampli‐tools (Version 1.0) (ed. Zenodo).

[ece34662-bib-0049] Krediet, C. J. , Ritchie, K. B. , Paul, V. J. , & Teplitski, M. (2013). Coral‐associated micro‐organisms and their roles in promoting coral health and thwarting diseases. Proceedings of the Royal Society B: Biological Sciences, 280(1755), 20122328–20122328. 10.1098/rspb.2012.2328. PMC3574386 23363627

[ece34662-bib-0050] Kwiatkowski, L. , Cox, P. , Halloran, P. R. , Mumby, P. J. , & Wiltshire, A. J. (2015). Coral bleaching under unconventional scenarios of climate warming and ocean acidification. Nature Climate Change, 5, 777 10.1038/nclimate2655.

[ece34662-bib-0051] LaJeunesse, T. (2002). Diversity and community structure of symbiotic dinoflagellates from Caribbean coral reefs. Marine Biology, 141, 387–400. 10.1007/s00227-002-0829-2.

[ece34662-bib-0052] LaJeunesse, T. C. , Bhagooli, R. , Hidaka, M. , deVantier, L. , Done, T. , Schmidt, G. W. , … Hoegh‐Guldberg, O. (2004). Closely related Symbiodinium spp. differ in relative dominance in coral reef host communities across environmental, latitudinal and biogeographic gradients. Marine Ecology Progress Series, 284, 147–161. 10.3354/meps284147.

[ece34662-bib-0053] LaJeunesse, T. C. , Loh, W. K. W. , van Woesik, R. , Hoegh‐Guldberg, O. , Schmidt, G. W. , & Fitt, W. K. . (2003). Low symbiont diversity in southern Great Barrier Reef corals, relative to those of the Caribbean. Limnology and Oceanography, 48, 2046–2054. 10.4319/lo.2003.48.5.2046.

[ece34662-bib-0054] LaJeunesse, T. C. , Parkinson, J. E. , Gabrielson, P. W. , Jeong, H. J. , Reimer, J. D. , Voolstra, C. R. , & Santos, S. R. (2018). Systematic revision of Symbiodiniaceae highlights the antiquity and diversity of coral endosymbionts. Current Biology, 28, 2570–2580. 10.1016/j.cub.2018.07.008. 30100341

[ece34662-bib-0055] LaJeunesse, T. C. , Pettay, D. T. , Sampayo, E. M. , Phongsuwan, N. , Brown, B. , Obura, D. O. , … Fitt, W. K. (2010). Long‐standing environmental conditions, geographic isolation and host–symbiont specificity influence the relative ecological dominance and genetic diversification of coral endosymbionts in the genus *Symbiodinium* . Journal of Biogeography, 37, 785–800.

[ece34662-bib-0056] LaJeunesse, T. C. , Smith, R. T. , Finney, J. , & Oxenford, H. (2009). Outbreak and persistence of opportunistic symbiotic dinoflagellates during the 2005 Caribbean mass coral ‘bleaching’ event. Proceedings of the Royal Society B: Biological Sciences, 276, 4139–4148. 10.1098/rspb.2009.1405. PMC2821356 19740874

[ece34662-bib-0057] LaJeunesse, T. C. , Wham, D. C. , Pettay, D. T. , Parkinson, J. E. , Keshavmurthy, S. , & Chen, C. A. (2014). Ecologically differentiated stress‐tolerant endosymbionts in the dinoflagellate genus Symbiodinium (Dinophyceae) Clade D are different species. Phycologia, 53, 305–319.

[ece34662-bib-0058] Li, J. , Chen, Q. , Long, L.‐J. , Dong, J.‐D. , Yang, J. , & Zhang, S. (2014). Bacterial dynamics within the mucus, tissue and skeleton of the coral *Porites lutea* during different seasons. Scientific Reports, 4, 7320 10.1038/srep07320. 25475855 PMC4256709

[ece34662-bib-0059] Liang, J. , Yu, K. , Wang, Y. , Huang, X. , Huang, W. , Qin, Z. , … Wu, Z. (2017). Distinct Bacterial communities associated with massive and branching Scleractinian corals and potential linkages to coral susceptibility to thermal or cold stress. Frontiers in Microbiology, 8, 979 10.3389/fmicb.2017.00979. 28642738 PMC5462945

[ece34662-bib-0060] Littman, R. A. , Bourne, D. G. , & Willis, B. L. (2010). Responses of coral‐associated bacterial communities to heat stress differ with Symbiodinium type on the same coral host. Molecular Ecology, 19, 1978–1990. 20529072 10.1111/j.1365-294X.2010.04620.x

[ece34662-bib-0061] Littman, R. , Willis, B. L. , & Bourne, D. G. (2011). Metagenomic analysis of the coral holobiont during a natural bleaching event on the Great Barrier Reef. Environmental Microbiology Reports, 3, 651–660. 10.1111/j.1758-2229.2010.00234.x. 23761353

[ece34662-bib-0062] Littman, R. A. , Willis, B. L. , Pfeffer, C. , & Bourne, D. G. (2009). Diversities of coral‐associated bacteria differ with location, but not species, for three acroporid corals on the Great Barrier Reef. FEMS Microbiology Ecology, 68, 152–163. 10.1111/j.1574-6941.2009.00666.x. 19302548

[ece34662-bib-0063] Liu, G. , Heron, S. , Eakin, C. , Muller‐Karger, F. , Vega‐Rodriguez, M. , Guild, L. , … Lynds, S. (2014). Reef‐scale thermal stress monitoring of coral ecosystems: New 5‐km global products from NOAA coral reef watch. Remote Sensing, 6, 11579 10.3390/rs61111579.

[ece34662-bib-0064] Long, M. H. , Rheuban, J. E. , Berg, P. , & Zieman, J. C. (2012). A comparison and correction of light intensity loggers to photosynthetically active radiation sensors. Limnology and Oceanography: Method, 10, 416–424. 10.4319/lom.2012.10.416

[ece34662-bib-0065] Magoč, T. , & Salzberg, S. L. (2011). FLASH: Fast length adjustment of short reads to improve genome assemblies. Bioinformatics, 27, 2957–2963. 10.1093/bioinformatics/btr507. 21903629 PMC3198573

[ece34662-bib-0066] McClanahan, T. R. (2017). Changes in coral sensitivity to thermal anomalies. Marine Ecology Progress Series, 570, 71–85. 10.3354/meps12150.

[ece34662-bib-0067] McClanahan, T. R. , Ateweberhan, M. , Darling, E. S. , Graham, N. A. J. , & Muthiga, N. A. (2014). Biogeography and Change among Regional Coral Communities across the Western Indian Ocean. PLoS ONE, 9, e93385 10.1371/journal.pone.0093385. 24718371 PMC3981710

[ece34662-bib-0068] McClanahan, T. R. , Ateweberhan, M. , Graham, N. A. J. , Wilson, S. K. , Sebastián, C. R. , Guillaume, M. M. M. , & Bruggemann, J. H. (2007). Western Indian Ocean coral communities: Bleaching responses and susceptibility to extinction. Marine Ecology Progress Series, 337, 1–13. 10.3354/meps337001.

[ece34662-bib-0069] McDevitt‐Irwin, J. M. , Baum, J. K. , Garren, M. , & Vega Thurber, R. L. (2017). Responses of coral‐associated bacterial communities to local and global stressors. Frontiers in Marine Science, 4, 262 10.3389/fmars.2017.00262

[ece34662-bib-0070] Meyer, J. L. , Paul, V. J. , & Teplitski, M. (2014). Community shifts in the surface microbiomes of the coral *Porites astreoides* with unusual lesions. PLoS ONE, 9, e100316 10.1371/journal.pone.0100316. 24937478 PMC4061089

[ece34662-bib-0071] Morrow, K. M. , Moss, A. G. , Chadwick, N. E. , & Liles, M. R. (2012). Bacterial associates of two Caribbean coral species reveal species‐specific distribution and geographic variability. Applied and Environmental Microbiology, 78, 6438–6449. 10.1128/AEM.01162-12. 22773636 PMC3426691

[ece34662-bib-0072] Morrow, K. M. , Muller, E. , & Lesser, M. P. (2018). How does the coral microbiome cause, respond to, or modulate the bleaching process? In van Oppen M. J. H., & Lough J. M. (Eds.), Coral Bleaching: Patterns, Processes, Causes and Consequences (pp. 153–188). Cham, Switzerland: Springer International Publishing.

[ece34662-bib-0073] Neave, M. J. , Apprill, A. , Ferrier‐Pagès, C. , & Voolstra, C. R. (2016). Diversity and function of prevalent symbiotic marine bacteria in the genus *Endozoicomonas* . Applied Microbiology and Biotechnology, 100, 8315–8324. 10.1007/s00253-016-7777-0. 27557714 PMC5018254

[ece34662-bib-0074] Neave, M. J. , Michell, C. T. , Apprill, A. , & Voolstra, C. R. (2017b). *Endozoicomonas* genomes reveal functional adaptation and plasticity in bacterial strains symbiotically associated with diverse marine hosts. Scientific Reports, 7, 40579. 28094347 10.1038/srep40579 PMC5240137

[ece34662-bib-0075] Neave, M. J. , Rachmawati, R. , Xun, L. , Michell, C. T. , Bourne, D. G. , Apprill, A. , & Voolstra, C. R. (2017a). Differential specificity between closely related corals and abundant *Endozoicomonas* endosymbionts across global scales. The ISME Journal, 11, 186–200. 27392086 10.1038/ismej.2016.95 PMC5335547

[ece34662-bib-0076] Oliver, T. A. , & Palumbi, S. R. (2009). Distributions of stress‐resistant coral symbionts match environmental patterns at local but not regional scales. Marine Ecology Progress Series, 378, 93–103.

[ece34662-bib-0077] Pantos, O. , Bongaerts, P. , Dennis, P. G. , Tyson, G. W. , & Hoegh‐Guldberg, O. (2015). Habitat‐specific environmental conditions primarily control the microbiomes of the coral *Seriatopora hystrix* . The ISME Journal, 9, 1916–1927. 10.1038/ismej.2015.3. 25668159 PMC4542040

[ece34662-bib-0078] Pettay, D. T. , Wham, D. C. , Smith, R. T. , Iglesias‐Prieto, R. , & LaJeunesse, T. C. (2015). Microbial invasion of the Caribbean by an Indo‐Pacific coral zooxanthella. Proceedings of the National Academy of Sciences of the United States of America, 112, 7513–7518. 10.1073/pnas.1502283112. 26034268 PMC4475936

[ece34662-bib-0079] Pogoreutz, C. , Rädecker, N. , Cárdenas, A. , Gärdes, A. , Voolstra, C. R. , & Wild, C. (2017). Sugar enrichment provides evidence for a role of nitrogen fixation in coral bleaching. Global Change Biology, 9, 3838–3848. 10.1111/gcb.13695. 28429531

[ece34662-bib-0080] Pogoreutz, C. , Rädecker, N. , Cárdenas, A. , Gärdes, A. , Wild, C. , & Voolstra, C. R. (2018). Dominance of *Endozoicomonas* bacteria throughout coral bleaching and mortality suggests structural inflexibility of the *Pocillopora verrucosa* microbiome. Ecology and Evolution, 8, 2240–2252. 29468040 10.1002/ece3.3830 PMC5817147

[ece34662-bib-0081] Pootakham, W. , Mhuantong, W. , Yoocha, T. , Putchim, L. , Sonthirod, C. , Naktang, C. , … Tangphatsornruang, S. (2017). High resolution profiling of coral‐associated bacterial communities using full‐length 16S rRNA sequence data from PacBio SMRT sequencing system. Scientific Reports, 7, 2774 10.1038/s41598-017-03139-4. 28584301 PMC5459821

[ece34662-bib-0082] Putnam, H. M. , Barott, K. L. , Ainsworth, T. D. , & Gates, R. D. (2017). The vulnerability and resilience of reef‐building corals. Current Biology, 27, R528–R540. 10.1016/j.cub.2017.04.047. 28586690

[ece34662-bib-0083] Raina, J.‐B. , Tapiolas, D. M. , Foret, S. , Lutz, A. , Abrego, D. , Ceh, J. , … Motti, C. A. (2013). DMSP biosynthesis by an animal and its role in coral thermal stress response. Nature, 502, 677–680. 10.1038/nature12677. 24153189

[ece34662-bib-0084] Reisch, C. R. , Moran, M. A. , & Whitman, W. B. (2011). Bacterial catabolism of dimethylsulfoniopropionate (DMSP). Frontiers in Microbiology, 2, 172 10.3389/fmicb.2011.00172. 21886640 PMC3155054

[ece34662-bib-0085] Reshef, L. , Koren, O. , Loya, Y. , Zilber‐Rosenberg, I. , & Rosenberg, E. (2006). The coral probiotic hypothesis. Environmental Microbiology, 8, 2068–2073. 10.1111/j.1462-2920.2006.01148.x. 17107548

[ece34662-bib-0086] Roder, C. , Arif, C. , Bayer, T. , Aranda, M. , Daniels, C. , Shibl, A. , … Voolstra, C. R. (2013). Bacterial profiling of White Plague Disease in a comparative coral species framework. The ISME Journal, 8, 31 10.1038/ismej.2013.127. 23924783 PMC3869008

[ece34662-bib-0087] Roder, C. , Arif, C. , Daniels, C. , Weil, E. , & Voolstra, C. R. (2014). Bacterial profiling of White Plague Disease across corals and oceans indicates a conserved and distinct disease microbiome. Molecular Ecology, 23, 965–974. 10.1111/mec.12638. 24350609 PMC4285310

[ece34662-bib-0088] Roder, C. , Bayer, T. , Aranda, M. , Kruse, M. , & Voolstra, C. R. (2015). Microbiome structure of the fungid coral *Ctenactis echinata* aligns with environmental differences. Molecular Ecology, 24, 3501–3511. 26018191 10.1111/mec.13251 PMC4736464

[ece34662-bib-0089] Rognes, T. , Flouri, T. , Nichols, B. , Quince, C. , & Mahé, F. (2016). VSEARCH: A versatile open source tool for metagenomics. PeerJ, 4, e2584 10.7717/peerj.2584. 27781170 PMC5075697

[ece34662-bib-0090] Rohwer, F. , Breitbart, M. , Jara, J. , Azam, F. , & Knowlton, N. (2001). Diversity of bacteria associated with the Caribbean coral *Montastraea franksi* . Coral Reefs, 20, 85–91. 10.1007/s003380100138.

[ece34662-bib-0091] Rohwer, F. , Seguritan, V. , Azam, F. , & Knowlton, N. (2002). Diversity and distribution of coral‐associated bacteria. Marine Ecology Progress Series, 243, 1–10. 10.3354/meps243001.

[ece34662-bib-0092] Rosenberg, E. , Koren, O. , Reshef, L. , Efrony, R. , & Zilber‐Rosenberg, I. (2007). The role of microorganisms in coral health, disease and evolution. Nature Reviews Microbiology, 5, 355 10.1038/nrmicro1635. 17384666

[ece34662-bib-0093] Röthig, T. , Ochsenkühn, M. A. , Roik, A. , van der Merwe, R. , & Voolstra, C. R. (2016). Long‐term salinity tolerance is accompanied by major restructuring of the coral bacterial microbiome. Molecular Ecology, 25, 1308–1323. 10.1111/mec.13567. 26840035 PMC4804745

[ece34662-bib-0094] Rouzé, H. , Lecellier, G. , Saulnier, D. , & Berteaux‐Lecellier, V. (2016). Symbiodinium clades A and D differentially predispose *Acropora cytherea* to disease and Vibrio spp. colonization. Ecology and Evolution, 6, 560–572. 26843939 10.1002/ece3.1895 PMC4729262

[ece34662-bib-0095] Salter, S. J. , Cox, M. J. , Turek, E. M. , Calus, S. T. , Cookson, W. O. , Moffatt, M. F. , … Walker, A. W. (2014). Reagent and laboratory contamination can critically impact sequence‐based microbiome analyses. BMC Biology, 12, 87 10.1186/s12915-014-0087-z 25387460 PMC4228153

[ece34662-bib-0096] Schloss, P. D. , Westcott, S. L. , Ryabin, T. , Hall, J. R. , Hartmann, M. , Hollister, E. B. , … Weber, C. F. (2009). Introducing mothur: Open‐source, platform‐independent, community‐supported software for describing and comparing microbial communities. Applied and Environmental Microbiology, 75, 7537–7541. 10.1128/AEM.01541-09. 19801464 PMC2786419

[ece34662-bib-0097] Silverstein, R. N. , Correa, A. M. S. , LaJeunesse, T. C. , & Baker, A. C. (2011). Novel algal symbiont (Symbiodinium spp.) diversity in reef corals of Western Australia. Marine Ecology Progress Series, 422, 63–75.

[ece34662-bib-0098] Smith, E. G. , Ketchum, R. N. , & Burt, J. A. (2017). Host specificity of Symbiodinium variants revealed by an ITS2 metahaplotype approach. The ISME Journal, 11, 1500–1503. 10.1038/ismej.2016.206. 28211848 PMC5437344

[ece34662-bib-0099] Smith, E. G. , Vaughan, G. O. , Ketchum, R. N. , McParland, D. , & Burt, J. A. (2017). Symbiont community stability through severe coral bleaching in a thermally extreme lagoon. Scientific Reports, 7, 2428 10.1038/s41598-017-01569-8. 28546553 PMC5445074

[ece34662-bib-0100] Smith, L. W. , Wirshing, H. H. , Baker, A. C. , & Birkeland, C. (2008). Environmental versus genetic influences on growth rates of the corals *Pocillopora eydouxi* and *Porites lobata* (Anthozoa: Scleractinia). Pacific Science, 62, 57–69.

[ece34662-bib-0101] Suggett, D. J. , Warner, M. E. , & Leggat, W. (2017). Symbiotic dinoflagellate functional diversity mediates coral survival under ecological crisis. Trends in Ecology & Evolution, 32, 735–745. 10.1016/j.tree.2017.07.013. 28843439

[ece34662-bib-0102] Sweet, M. J. , & Bulling, M. T. (2017). On the importance of the microbiome and pathobiome in coral health and disease. Frontiers in Marine Science, 4, 9 10.3389/fmars.2017.00009.

[ece34662-bib-0103] Thornhill, D. J. , Lewis, A. M. , Wham, D. C. , & LaJeunesse, T. C. (2014). Host‐specialist lineages dominate the adaptive radiation of reef coral endosymbionts. Evolution, 68, 352–367. 10.1111/evo.12270. 24134732

[ece34662-bib-0104] Torda, G. , Donelson, J. M. , Aranda, M. , Barshis, D. J. , Bay, L. , Berumen, M. L. , … Munday, P. L. (2017). Rapid adaptive responses to climate change in corals. Nature Climate Change, 7, 627 10.1038/nclimate3374.

[ece34662-bib-0105] Tout, J. , Siboni, N. , Messer, L. F. , Garren, M. , Stocker, R. , Webster, N. S. , … Seymour, J. R. (2015). Increased seawater temperature increases the abundance and alters the structure of natural Vibrio populations associated with the coral *Pocillopora damicornis* . Frontiers in Microbiology, 6, 432. 10.3389/fmicb.2015.00432. 26042096 PMC4435422

[ece34662-bib-0106] Ulstrup, K. E. , & Van Oppen, M. J. H. (2003). Geographic and habitat partitioning of genetically distinct zooxanthellae (*Symbiodinium*) in Acropora corals on the Great Barrier Reef. Molecular Ecology, 12, 3477–3484. 10.1046/j.1365-294X.2003.01988.x 14629362

[ece34662-bib-0107] Vega Thurber, R. L. , Burkepile, D. E. , Fuchs, C. , Shantz, A. A. , McMinds, R. , & Zaneveld, J. R. (2014). Chronic nutrient enrichment increases prevalence and severity of coral disease and bleaching. Global Change Biology, 20, 544–554. 10.1111/gcb.12450. 24277207

[ece34662-bib-0108] Vega Thurber, R. , Willner‐Hall, D. , Rodriguez‐Mueller, B. , Desnues, C. , Edwards, R. A. , Angly, F. , … Rohwer, F. (2009). Metagenomic analysis of stressed coral holobionts. Environmental Microbiology, 11, 2148–2163. 10.1111/j.1462-2920.2009.01935.x. 19397678

[ece34662-bib-0109] Wilson, S. K. , Graham, N. A. , Fisher, R. , Robinson, J. , Nash, K. , Chong‐Seng, K. , … Quatre, R. (2012). Effect of macroalgal expansion and marine protected areas on coral recovery following a climatic disturbance. Conservation Biology, 26, 995–1004. 10.1111/j.1523-1739.2012.01926.x. 22971046

[ece34662-bib-0110] Yang, C. S. , Chen, M. H. , Arun, A. B. , Chen, C. A. , Wang, J. T. , & Chen, W. M. (2010). *Endozoicomonas montiporae* sp. nov., isolated from the encrusting pore coral Montipora aequituberculata. International Journal of Systematic and Evolutionary Microbiology, 60, 1158–1162. 10.1099/ijs.0.014357-0. 19666790

[ece34662-bib-0111] Yang, S.‐Y. , Keshavmurthy, S. , Obura, D. , Sheppard, C. R. C. , Visram, S. , & Chen, C. A. (2012). Diversity and Distribution of symbiodinium associated with seven common coral species in the Chagos Archipelago, Central Indian Ocean. Plos One, 7, e35836. 22567113 10.1371/journal.pone.0035836 PMC3342320

[ece34662-bib-0112] Ziegler, M. , Eguíluz, V. M. , Duarte, C. M. , & Voolstra, C. R. (2017a). Rare symbionts may contribute to the resilience of coral–algal assemblages. The ISME Journal, 12, 161–172. 29192903 10.1038/ismej.2017.151 PMC5739009

[ece34662-bib-0113] Ziegler, M. , Roik, A. , Porter, A. , Zubier, K. , Mudarris, M. S. , Ormond, R. , & Voolstra, C. R. (2016). Coral microbial community dynamics in response to anthropogenic impacts near a major city in the central Red Sea. Marine Pollution Bulletin, 105, 629–640. 10.1016/j.marpolbul.2015.12.045. 26763316

[ece34662-bib-0114] Ziegler, M. , Seneca, F. O. , Yum, L. K. , Palumbi, S. R. , & Voolstra, C. R. (2017b). Bacterial community dynamics are linked to patterns of coral heat tolerance. Nature Communications, 8, 14213. 10.1038/ncomms14213 PMC5309854 28186132

[ece34662-bib-0115] Zinke, J. , Gilmour, J. P. , Fisher, R. , Puotinen, M. , Maina, J. , Darling, E. , … Wilson, S. K. (2018). Gradients of disturbance and environmental conditions shape coral community structure for south‐eastern Indian Ocean reefs. Diversity and Distributions, 1, 1–16. 10.1111/ddi.12714.

