# Supplementary material for: Coral microbiome diversity reflects mass coral bleaching susceptibility during the 2016 El Niño heat wave
Source: Ecol Evol. 2019 Jan 17;9(3):938–56. doi: 10.1002/ece3.4662 (PMC6374667; doi:10.1002/ece3.4662)
Supplement: Supplementary file 1 [file ECE3-9-938-s001.docx]

**Supplementary information**

**Coral microbiome diversity reflects mass coral bleaching susceptibility during the 2016 El Niño heat wave**

Stephanie G. Gardner^1^, Emma F. Camp^1^, David J. Smith^2^*, Tim Kahlke^1^, Eslam O. Osman^2,3^, Gilberte Gendron^4^, Benjamin C.C. Hume^5^, Claudia Pogoreutz^5^, Christian R. Voolstra^5^, David J. Suggett^1^

**Tables**

**Supplementary Table 1** a) Permutational MANOVA (PERMANOVA) using Bray-Curtis dissimilarity to compare differences in Symbiodiniaceae ITS2 type profiles between species, site and coral condition for *Acropora gemmifera, Acropora muricata, Coelastrea aspera* and *Porites lutea* at East Bay and Praslin in the Seychelles. Statistically significant interactions between species are shown in red. Data were fourth-root transformed b) Summary of ANOSIM pairwise tests for Symbiodiniaceae ITS2 type profile associated with the corals *Acropora gemmifera, Acropora muricata, Coelastrea aspera* and *Porites lutea* at East Bay and Praslin in the Seychelles. Statistically significant interactions between species are shown in red. Global R = 0.25, P = 0.001.

1. PERMANOVA

| **Source** | **df** | **SS** | **MS** | **Pseudo-F** | **P value** | **Unique perms** |
| --- | --- | --- | --- | --- | --- | --- |
| Species | 3 | 51216 | 17072 | 6.3267 | 0.001 | 997 |
| Site | 1 | 4008.1 | 4008.1 | 1.4854 | 0.176 | 998 |
| Coral condition | 1 | 6466.9 | 6466.9 | 2.3966 | 0.074 | 999 |
| Species × Site | 3 | 12818 | 4272.6 | 1.5834 | 0.139 | 999 |
| Species × Condition | 3 | 14724 | 4908 | 1.8188 | 0.057 | 999 |
| Site × Condition | 1 | 3340.9 | 3340.9 | 1.2381 | 0.250 | 997 |
| Species × Site × Condition | 3 | 11809 | 3936.4 | 1.4588 | 0.164 | 998 |
| Residual | 32 | 57767 | 2698.4 |  |  |  |
| Total | 47 | 1.91E+05 |  |  |  |  |

b) ANOSIM

| Pairwise Tests |  |  |  |  |  |
| --- | --- | --- | --- | --- | --- |
| **Groups** | **R Statistic** | **P value** | **Possible perms** | **Actual perms** | **Number >= observed** |
| *Coelastrea aspera, Acropora muricata* | 0.028 | 0.2180 | 1352078 | 9999 | 2181 |
| *Coelastrea aspera, Acropora gemmifera* | 0.078 | 0.0830 | 1352078 | 9999 | 833 |
| *Coelastrea aspera, Porites lutea* | 0.396 | 0.0001 | 1352078 | 9999 | 0 |
| *Acropora muricata, Acropora gemmifera* | 0.014 | 0.2770 | 1352078 | 9999 | 2768 |
| *Acropora muricata, Porites lutea* | 0.436 | 0.0001 | 1352078 | 9999 | 0 |
| *Acropora gemmifera, Porites lutea* | 0.513 | 0.0001 | 1352078 | 9999 | 0 |

**Supplementary Table 2** Summary of SIMPER analysis based on Bray-Curtis dissimilarity matrix, showing the average abundance (Av.Abund), average similarity (Av.Sim), contribution (contrib%) and cumulative percent (cum%) of the Symbiodiniaceae ITS2 type profiles contributing to the similarity between coral colonies across all sites (East Bay and Praslin) and condition (bleached and unbleached) for *Acropora gemmifera, Acropora muricata, Coelastrea aspera* and *Porites lutea*.

|  | | **Av.Abund** | | **Av.Sim** | | **Contrib%** | | **Cum.%** | |
| --- | --- | --- | --- | --- | --- | --- | --- | --- | --- |
| ***Acropora gemmifera*** | |  | |  | |  | |  | |
| Average similarity: 40.99 % | |  | |  | |  | |  | |
| C3z-C3-C3.10-C3bq | | 88.04 | | 21.3 | | 51.96 | | 51.96 | |
| C3z/C3-C3.10-C3an | | 86.96 | | 19.35 | | 47.22 | | 99.18 | |
| D1-D4-D4c-D1c-D17d-D17e-D17c | | 9.34 | | 0.34 | | 0.82 | | 100 | |
| ***Acropora muricata*** | |  | |  | |  | |  | |
| Average similarity: 26.13 % | |  | |  | |  | |  | |
| C3z/C3-C3.10-C3an | | 83 | | 19.19 | | 73.43 | | 73.43 | |
| C3z-C3-C3.10-C3bq | | 47.78 | | 5.49 | | 21.03 | | 94.46 | |
| D1-D2.2-D1m-D4-D2c | | 29.49 | | 1.33 | | 5.09 | | 99.55 | |
| D1-D2.2-D1m-D4 | | 7.58 | | 0.12 | | 0.45 | | 100 | |
| ***Coelastrea aspera*** | |  | |  | |  | |  | |
| Average similarity: 7.09 % | |  | |  | |  | |  | |
| D1-D4-D4c-D2-D4f | | 22.64 | | 2.03 | | 28.62 | | 28.62 | |
| C3z/C3-C3.10-C3an | | 30.28 | | 2 | | 28.24 | | 56.86 | |
| C3z-C3-C3.10-C3bq | | 20.07 | | 1.53 | | 21.56 | | 78.42 | |
| D1-D4-D2-D4c-D4f-D6 | | 27.68 | | 1.07 | | 15.14 | | 93.57 | |
| C3u | | 9.56 | | 0.27 | | 3.76 | | 97.32 | |
| D1-D4-D4c-D2-D6-D1c | | 8.22 | | 0.19 | | 2.68 | | 100 | |
| ***Porites lutea*** | |  | |  | |  | |  | |
| Average similarity: 52.22 % | |  | |  | |  | |  | |
| C15-C15ad | | 120.32 | | 47.84 | | 91.61 | | 91.61 | |
| C15 | | 40.79 | | 3 | | 5.75 | | 97.36 | |
| D9 | | 12.11 | | 0.93 | | 1.78 | | 99.14 | |
| D4 | | 5.17 | | 0.33 | | 0.63 | | 99.77 | |
| C3z-C3-C3.10-C3bq | | 3.4 | | 0.12 | | 0.23 | | 100 | |
|  | |  | |  | |  | |  | |
|  |  | |  | |  | |  | |  |

**Supplementary Table 3** a) Permutational MANOVA (PERMANOVA) using Bray-Curtis dissimilarity to compare differences in bacterial community composition between species, site and coral condition for *Acropora gemmifera, Acropora muricata, Coelastrea aspera* and *Porites lutea* at East Bay and Praslin in the Seychelles. Statistically significant interactions between species are shown in red. Data were fourth-root transformed. b) Summary of ANOSIM pairwise tests for bacterial community compositions (based on 16S rRNA gene sequences) associated with the corals *Acropora gemmifera, Acropora muricata, Coelastrea aspera* and *Porites lutea* at East Bay and Praslin in the Seychelles. Statistically significant interactions between species are shown in red. Global R = 0.376, P = 0.0001.

1. PERMANOVA

| **Source** | **df** | **SS** | **MS** | **Pseudo-F** | **P value** | **Unique perms** |
| --- | --- | --- | --- | --- | --- | --- |
| Species | 3 | 26836 | 8945.3 | 4.9553 | 0.0001 | 9921 |
| Site | 1 | 2352 | 2352 | 1.3029 | 0.1959 | 9917 |
| Coral condition | 1 | 1894.9 | 1894.9 | 1.0497 | 0.3287 | 9904 |
| Species × Site | 3 | 5813 | 1937.7 | 1.0734 | 0.3371 | 9890 |
| Species × Condition | 3 | 7018.3 | 2339.4 | 1.2959 | 0.1661 | 9888 |
| Site × Condition | 1 | 1762.3 | 1762.3 | 0.97625 | 0.3833 | 9903 |
| Species × Site × Condition | 3 | 5190.6 | 1730.2 | 0.95846 | 0.4838 | 9894 |
| Residual | 32 | 57767 | 1805.2 |  |  |  |
| Total | 47 | 1.09E+05 |  |  |  |  |

1. ANOSIM

| Pairwise Tests | **R Statistic** | **P value** | **Possible perms** | **Actual perms** | **Number >= observed** |
| --- | --- | --- | --- | --- | --- |
| Group |  |  |  |  |  |
| *Coelastrea aspera, Acropora muricata* | 0.388 | 0.0020 | 1352078 | 9999 | 14 |
| *Coelastrea aspera, Acropora gemmifera* | 0.411 | 0.0002 | 1352078 | 9999 | 1 |
| *Coelastrea aspera, Porites lutea* | 0.065 | 0.0940 | 1352078 | 9999 | 942 |
| *Acropora muricata, Acropora gemmifera* | 0.126 | 0.0240 | 1352078 | 9999 | 234 |
| *Acropora muricata, Porites lutea* | 0.670 | 0.0001 | 1352078 | 9999 | 0 |
| *Acropora gemmifera, Porites lutea* | 0.641 | 0.0001 | 1352078 | 9999 | 0 |

**Supplementary Table 4** a) Permutational MANOVA (PERMANOVA) and b) ANOSIM pairwise tests comparing differences in bacterial community composition once the most abundant families (Hahellaceae, Rhodospirillaceae and Rhodobacteraceae) were excluded. Statistically significant interactions between species are shown in red. Global R = 0.355, P = 0.0001.

1. PERMANOVA

| **Source** | **df** | **SS** | **MS** | **Pseudo-F** | **P value** | **Unique perms** |
| --- | --- | --- | --- | --- | --- | --- |
| Species | 3 | 29336 | 9778.7 | 5.1956 | 0.000 | 9900 |
| Site | 1 | 3226.4 | 3226.4 | 1.7142 | 0.057 | 9915 |
| Coral condition | 1 | 2861.8 | 2861.8 | 1.5205 | 0.095 | 9896 |
| Species × Site | 3 | 9200.4 | 3066.8 | 1.6294 | 0.020 | 9877 |
| Species × Condition | 3 | 6516.2 | 2172.1 | 1.1541 | 0.235 | 9886 |
| Site × Condition | 1 | 3135.5 | 3135.5 | 1.666 | 0.074 | 9926 |
| Species × Site × Condition | 3 | 6685.8 | 2228.6 | 1.1841 | 0.195 | 9872 |
| Residual | 32 | 60228 | 1882.1 |  |  |  |
| Total | 47 | 1.21E+05 |  |  |  |  |

1. ANOSIM

| Pairwise Tests | **R Statistic** | **P value** | **Possible perms** | **Actual perms** | **Number >= observed** |
| --- | --- | --- | --- | --- | --- |
| Group |  |  |  |  |  |
| *Coelastrea aspera, Acropora muricata* | 0.07 | 0.102 | 1352078 | 9999 | 1022 |
| *Coelastrea aspera, Acropora gemmifera* | 0.328 | 0.0005 | 1352078 | 9999 | 4 |
| *Coelastrea aspera, Porites lutea* | 0.652 | 0.0001 | 1352078 | 9999 | 0 |
| *Acropora muricata, Acropora gemmifera* | 0.317 | 0.003 | 1352078 | 9999 | 26 |
| *Acropora muricata, Porites lutea* | 0.675 | 0.0002 | 1352078 | 9999 | 1 |
| *Acropora gemmifera, Porites lutea* | 0.094 | 0.028 | 1352078 | 9999 | 280 |

**Supplementary Table 5** Summary of SIMPER analysis with Bray-Curtis dissimilarity, showing the average abundance (Av.Abund), average similarity (Av.Sim), contribution (contrib%) and cumulative percent (cum%) of the five top bacterial taxa (classified to family level) contributing to the similarity between coral colonies across both sites (East Bay and Praslin) and coral condition (bleached and unbleached) for *Acropora gemmifera, Acropora muricata, Coelastrea aspera* and *Porites lutea*.

| ***Acropora gemmifera*** |  |  |  |  |
| --- | --- | --- | --- | --- |
| Average similarity: 44.97 % |  |  |  |  |
| **Species** | **Av.Abund** | **Av.Sim** | **Contrib%** | **Cum.%** |
| Gammaproteobacteria;o_Oceanospirillales; f_Hahellaceae | 0.63 | 14.05 | 31.25 | 31.25 |
| Gammaproteobacteria; o_Alteromonadales; f_Alteromonadaceae | 0.16 | 2.16 | 4.81 | 36.07 |
| Alphaproteobacteria; o_ Rhodobacterales; f_Rhodobacteraceae | 0.16 | 1.93 | 4.29 | 40.35 |
| Bacilli; o_Bacillales; f_Staphylococcaceae | 0.11 | 1.92 | 4.28 | 44.63 |
| Deltaproteobacteria; o_Myxococcales; f_P3OB-42 | 0.18 | 1.61 | 3.58 | 48.21 |
| ***Acropora muricata*** |  |  |  |  |
| Average similarity: 41.82 % |  |  |  |  |
| **Species** | **Av.Abund** | **Av.Sim** | **Contrib%** | **Cum.%** |
| Gammaproteobacteria; o_Oceanospirillales; f_Hahellaceae | 0.65 | 18.76 | 44.87 | 44.87 |
| Gammaproteobacteria; o_Alteromonadales; f_Alteromonadaceae | 0.10 | 2.11 | 5.05 | 49.92 |
| Bacilli; o_Bacillales; f_Staphylococcaceae | 0.06 | 1.48 | 3.54 | 53.46 |
| Bacilli; o_Lactobacillales; f_Streptococcaceae | 0.04 | 1.11 | 2.66 | 56.12 |
| Gammaproteobacteria; o_Vibrionales; f_Vibrionaceae | 0.05 | 1.09 | 2.61 | 58.73 |
| ***Coelastrea aspera*** |  |  |  |  |
| Average similarity: 32.31 % |  |  |  |  |
| **Species** | **Av.Abund** | **Av.Sim** | **Contrib%** | **Cum.%** |
| Gammaproteobacteria; o_Oceanospirillales; f_Hahellaceae | 0.35 | 5.10 | 15.78 | 15.78 |
| Alphaproteobacteria; o_Rhodospirillales; f_Rhodospirillaceae | 0.30 | 2.16 | 6.68 | 22.46 |
| Alphaproteobacteria; o_Rhodobacterales; f_Rhodobacteraceae | 0.18 | 1.27 | 3.94 | 26.41 |
| Alphaproteobacteria; o_Rhizobiales; f_Phyllobacteriaceae | 0.10 | 0.85 | 2.62 | 29.03 |
| Gammaproteobacteria; o_Xanthomonadales; f_JTB255 marine benthic group | 0.10 | 0.76 | 2.34 | 31.37 |
| ***Porites lutea*** |  |  |  |  |
| Average similarity: 47.60 % |  |  |  |  |
| **Species** | **Av.Abund** | **Av.Sim** | **Contrib%** | **Cum.%** |
| Gammaproteobacteria; o_Oceanospirillales; f_Hahellaceae | 0.45 | 4.98 | 10.46 | 10.46 |
| Alphaproteobacteria; o_Rhodospirillales; f_Rhodospirillaceae | 0.35 | 3.89 | 8.17 | 18.63 |
| Alphaproteobacteria; o_Rhodobacterales; f_Rhodobacteraceae | 0.18 | 1.82 | 3.82 | 22.46 |
| Deltaproteobacteria; o_Desulfurellales; f_Desulfurellaceae | 0.13 | 1.07 | 2.24 | 24.70 |
| Gammaproteobacteria; o_KI89A clade; f_uncultured bacterium | 0.11 | 0.99 | 2.09 | 26.79 |

**Figures**

**
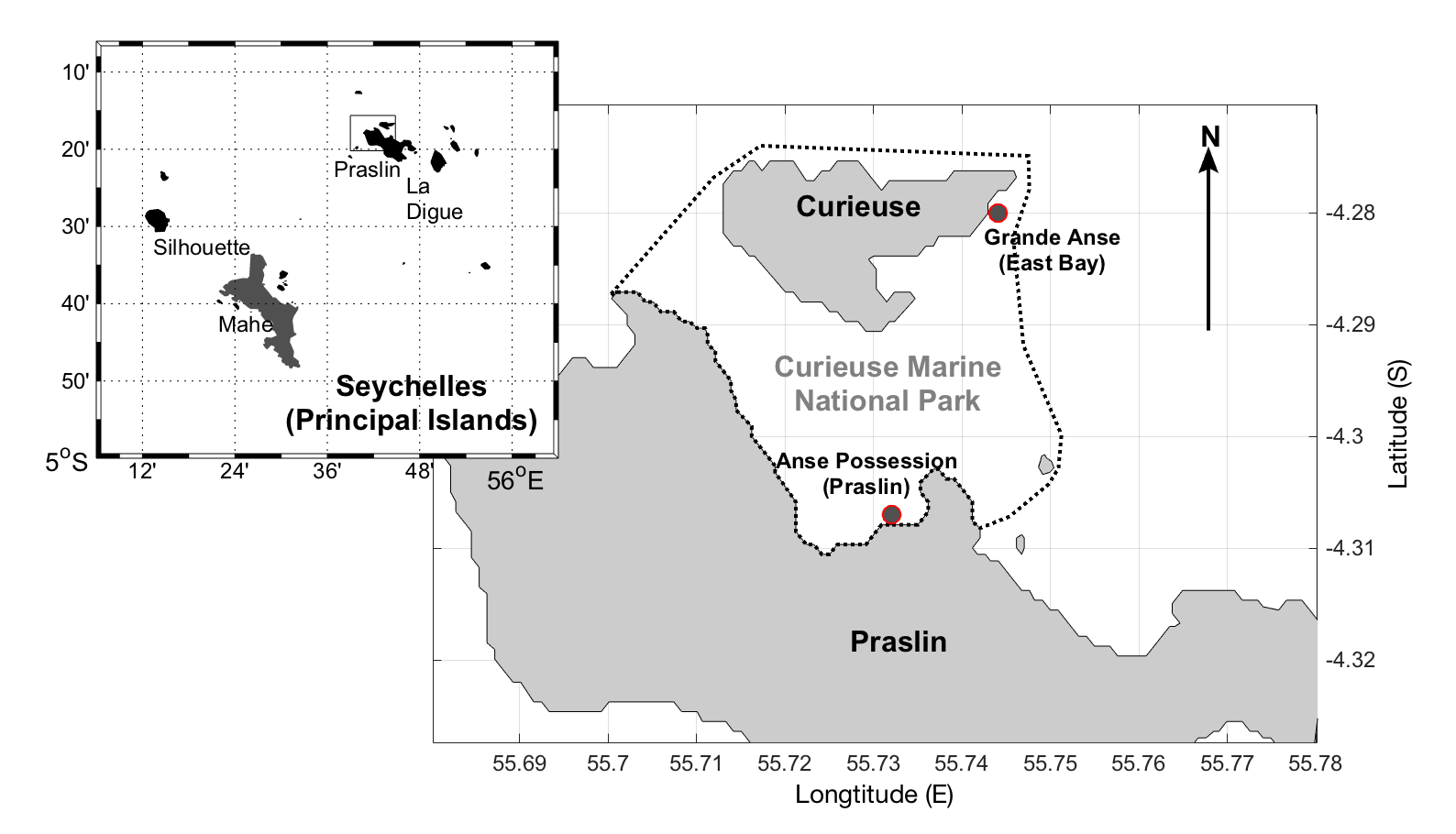
**

**Supplementary Fig. 1** Sampling sites of the turbid (Praslin; Anse Possession) and clear water (East Bay; Grande Anse) reefs within Curieuse Marine National Park (CMNP), Seychelles, Western Indian Ocean (WIO). Designated limits of CMNP are shown as the dot-dash line.

**
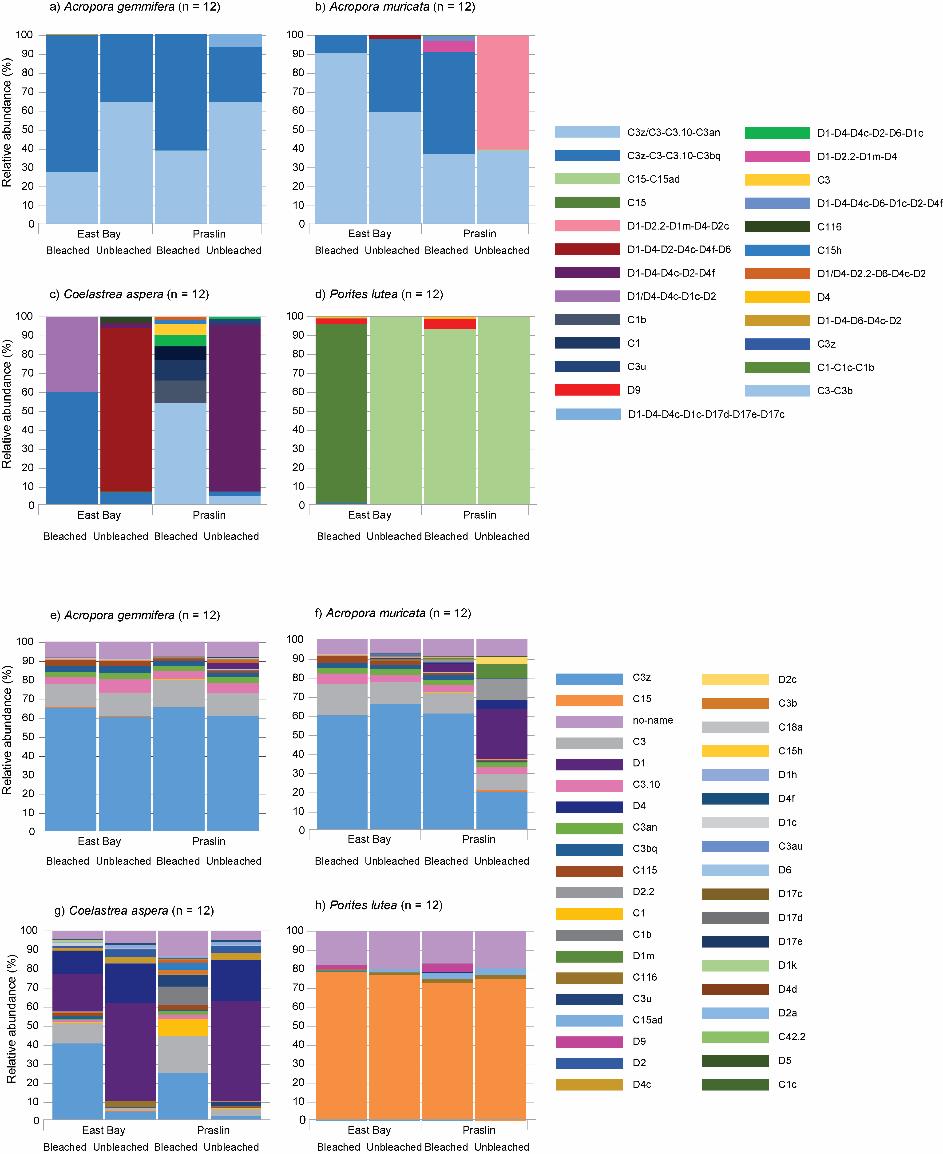
**

**Supplementary Fig. 2** Average relative abundance (%) of Symbiodiniaceae identified by a-d) ITS2 type profile and e-h) defining intragenomic variation (DIV) counts for bleached and unbleached corals as East Bay and Praslin for *Acropora gemmifera*, *Acropora muricata*, *Coelastrea aspera* and *Porites lutea* (n = 12 for each species). Colours represent different Symbiodiniaceae types.

**
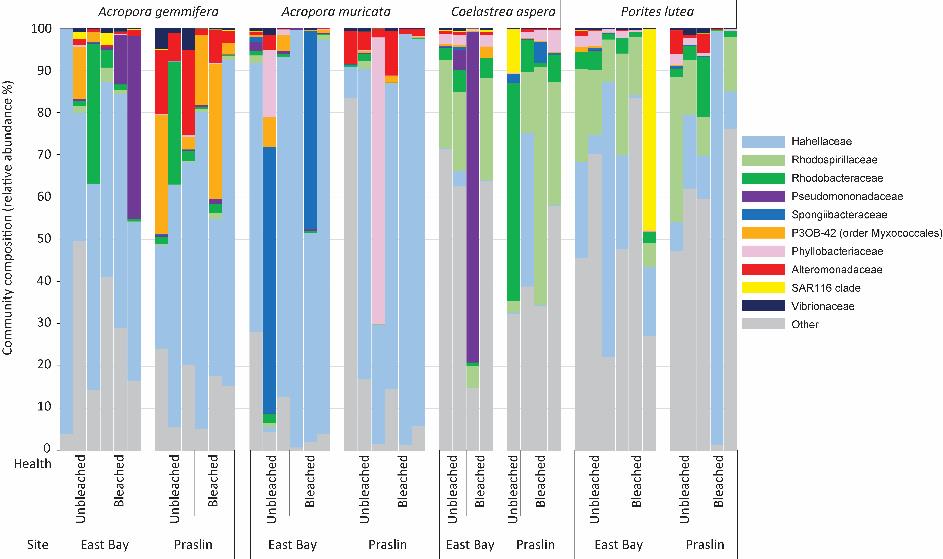
**

**Supplementary Fig. 3** Bacterial community composition for *Acropora gemmifera, Acropora muricata, Coelastrea aspera* and *Porites lutea* from two sites; East Bay and Praslin in the Seychelles. Depicted is a taxonomy stacked column plot to the phylogenetic level of family. Each colour represented one of the 15 most abundant families determined by rank abundance. Remaining families are grouped under the ‘Other’ category.
